# Role for the transcriptional activator ZRF1 in early metastatic events in breast cancer progression and endocrine resistance

**DOI:** 10.18632/oncotarget.25596

**Published:** 2018-06-19

**Authors:** Aysegül Kaymak, Sergi Sayols, Thaleia Papadopoulou, Holger Richly

**Affiliations:** ^1^ Laboratory of Molecular Epigenetics, Institute of Molecular Biology, Mainz, Germany; ^2^ Bioinformatics Core Facility, Institute of Molecular Biology, Mainz, Germany; ^3^ Department of Developmental and Stem Cell Biology, Institute Pasteur, Paris, France

**Keywords:** breast cancer, metastasis, cell invasion

## Abstract

Breast cancer is one of the most common malignancies among women which is often treated with hormone therapy and chemotherapy. Despite the improvements in detection and treatment of breast cancer, the vast majority of breast cancer patients are diagnosed with metastatic disease either at the beginning of the disease or later during treatment. Still, the molecular mechanisms causing a therapy resistant metastatic breast cancer are still elusive. In the present study we addressed the function of the transcriptional activator ZRF1 during breast cancer progression. We provide evidence that ZRF1 plays an essential role for the early metastatic events *in vitro* and acts like a tumor suppressor protein during the progression of breast invasive ductal carcinoma into a more advanced stage. Hence, depletion of ZRF1 results in the acquisition of metastatic behavior by facilitating the initiation of the metastatic cascade, notably for cell adhesion, migration and invasion. Furthermore absence of ZRF1 provokes endocrine resistance via misregulation of cell death and cell survival related pathways. Taken together, we have identified ZRF1 as an important regulator of breast cancer progression that holds the potential to be explored for new treatment strategies in the future.

## INTRODUCTION

Breast cancer (BC) is the most frequent carcinoma in females, and the second most common cause of cancer related mortality in women. Among all the parameters, estrogen is considered to be the main driver of breast cancer [1]. Approximately 70% of all breast cancers are estrogen receptor-positive (ER+) at the time of diagnosis. Hence, ER antagonists have been the standard therapy (also known as endocrine therapy) of choice for ER (+) breast cancers [2–4]. Tamoxifen (TAM) represents the first line treatment for both pre- and post-menopausal patients with ER (+) metastatic breast cancer. It is the classic member of SERMs (selective estrogen receptor modulators), which produce both estrogenic (breast and mammary tissue) and anti-estrogenic effects (bone and uterus) at the same time depending on the respective tissue [5–7]. Tamoxifen functions through competitive binding to ERs and by inhibiting estrogen dependent gene transcription, cell proliferation and tumor growth [8, 9]. As a prodrug, Tamoxifen is metabolized in the liver into active metabolites such as 4-hydroxytamoxifen (4-OHT) [10]. Another drug used in breast cancer treatment is ICI 182,780 (ICI), a selective estrogen receptor down-regulator (SERD), a pure anti-estrogen, which can down-regulate the ER expression levels and promote its proteasomal degradation [11]. ICI 182,780 lacks agonist effects on ER (+) tissues such as endometrium and uterus, and has an approximately 100 times greater binding affinity for ER [12–14]. Hence, in the clinic it is utilized for the treatment of Tamoxifen resistant hormone receptor positive metastatic breast cancer [15, 16]. However, the success of both drugs in the clinic is limited because as breast cancer cells evolve and grow in an estrogen independent manner they become resistant to endocrine therapy over time. Resistance to endocrine therapy is often accompanied by an upregulation of cell survival signaling and anti-apoptotic factors. Aberrant activation of the PI3K (Phosphatidylinositol-3 kinase)/AKT pathway, one of the key survival pathways, can lead to both estrogen independent growth [17] and resistance to cell death [18–20]. In addition to the PI3K/AKT pathway, increased expression of anti-apoptotic genes of the BCL-2 family also promotes endocrine resistance by decreasing the apoptotic response towards ER-antagonists [21, 22].

Nearly all breast cancer associated deaths are “generally” caused by the metastatic disease rather than the primary tumor itself. Formation of a metastatic disease requires a multi-step process known as the invasion-metastasis cascade [23–25]. The initial step of the invasion-metastasis cascade is the dissemination of cancer cells from primary tumors. One of the fundamental processes which enables this dissemination is epithelial-mesenchymal-transition (EMT). EMT is a biological process in which polarized epithelial cells undergo multiple biological changes that result in a mesenchymal cell phenotype with enhanced migratory capacity, invasiveness and resistance to apoptosis [26]. EMT normally occurs during embryogenesis but is hijacked by cancer cells to acquire mesenchymal features [27]. A critical molecular feature of EMT is the downregulation of the epithelial marker E-cadherin and the upregulation of mesenchymal markers such as N-cadherin, Vimentin and Fibronectin [26, 28]. As a complex mechanism, EMT is orchestrated by a series of master EMT-inducing transcription factors (EMT-TFs), specifically SNAIL, SLUG, TWIST, and ZEB1 [28, 29]. Activation of EMT programs results in the formation of carcinoma cells with mixed epithelial/mesenchymal phenotypes and a loss of cellular adhesion. Cancer metastasis proceeds with increased cell migration and invasion into the surrounding tissue [23, 30]. In this respect, understanding the molecular basis of the events that govern the progression of an early stage breast cancer towards a metastatic form is essential to avoid relapse and to ultimately decrease mortality.

Zuotin-related factor 1 (ZRF1) is a recently identified epigenetic regulator of gene transcription in stem cells and cancer [31, 32]. ZRF1 specifically binds to the histone H2A mono-ubiquitin mark at lysine 119 (H2AK119ub), a hallmark of gene silencing, which is set by Polycomb repressive complex 1 (PRC1). Upon recruitment to chromatin during cellular differentiation ZRF1 facilitates the activation of Polycomb repressed genes by displacing PRC1 from chromatin [32–34]. Hence, ZRF1 plays an essential role in the proper differentiation of ectoderm [35] and mesoderm derived tissues [36] during embryonic development. Apart from its function in differentiation ZRF1 plays a rather complex part in cancer progression. Expression and copy number alteration (CNA) analysis suggests a potential oncogenic role for ZRF1 as it is overexpressed in acute myeloid leukemia [37–39], chronic myeloid leukemia [40], chronic lymphocytic leukemia [41] and head and neck squamous cell carcinoma [42]. However, mechanistic studies suggest a dual role for ZRF1 as it can either work as tumor suppressor or induce carcinogenesis depending on the cellular context [31, 43]. Although ZRF1 plays an important role in many cancer types, its role in breast cancer is still poorly understood. According to the Oncomine database (www.oncomine.org), the expression of ZRF1 is reduced in breast cancer [44]. In accordance with this, a recent study analyzing tumor-associated antigens in the sera of breast cancer patients demonstrated that ZRF1 antigen and autoantibody response were suggested as a potential molecular marker of breast cancer. Moreover, the antibody response to the ZRF1 antigen was found to be higher in sera of patients with breast invasive ductal carcinoma particularly with less aggressive tumor phenotype [45]. On the contrary, another paper investigating the role of ZRF1 in breast cancer revealed that silencing of ZRF1 impedes survival of estrogen receptor positive MCF7 cells [68].

Here we report that ZRF1 keeps breast cancer progression at bay. ZRF1 depletion in breast invasive ductal carcinoma cells leads to events that mimic the early phases of metastasis *in vitro.* Knockdown of ZRF1 provokes the acquisition of metastatic traits by interfering important biological processes such as cell adhesion, cell migration and cell invasion. ZRF1 depleted cells contribute to the formation of tumor spheroids with an aggressive cancer phenotype in a 3D environment. Furthermore, these cells display endocrine resistance as a result of a disrupted balance between anti-apoptotic and pro-apoptotic genes and by the activation of the PI3K/AKT pathway. Our data suggest that ZRF1 is a potential novel target to be explored for new treatment strategies in breast cancer.

## RESULTS

### Depletion of ZRF1 has a mild effect on cell proliferation properties in MCF7 and T47D cells

**Figure 1:**
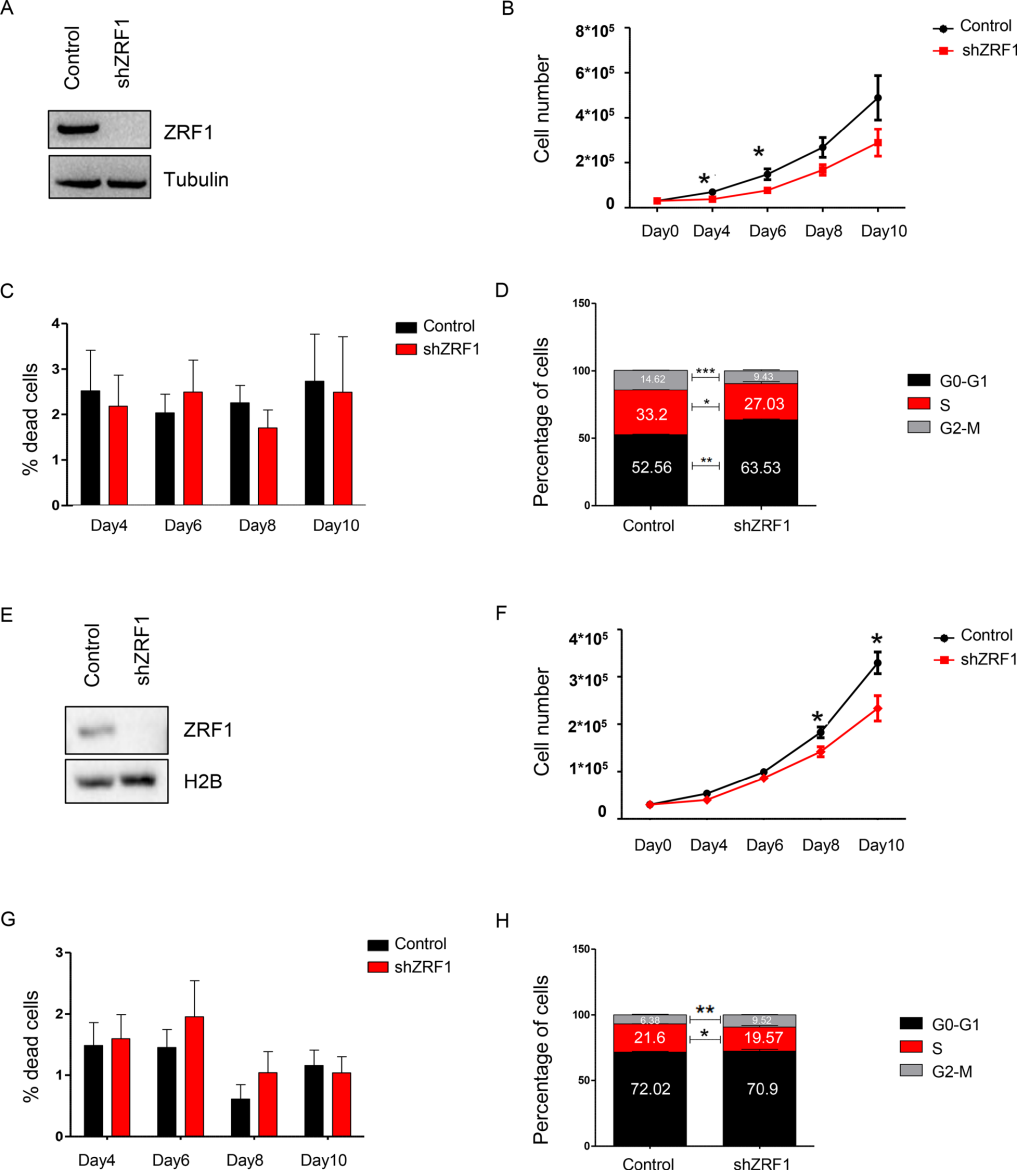
ZRF1 depletion has a mild effect on cell proliferation properties of MCF7 and T47D cells (**A**) Western blot for ZRF1 after viral induction of MCF7 cells. Alpha tubulin was used as a loading control. (**B**) Growth curve for MCF7 control and ZRF1 knockdown cells during 10 days. Data represent the average of three experiments, ± S.E.M. ^*^p < 0.5, calculated by two-tailed unpaired *t* test. (**C**) Cell death assay by trypan blue-positive cell count in control and ZRF1 depleted MCF7 cells during 10 days. Data represent the average of three experiments. (**D**) Flow cytometry analysis of cell cycle distribution of control and shZRF1 cells. Data represent the average of three experiments, ± S.E.M. ^*^p < 0.5, ^**^p < 0.01, ^***^p < 0.001 calculated by two-tailed unpaired *t* test. (**E**) Western blot for ZRF1 after viral induction of T47D cells. Histone H2B was used as a loading control. (**F**) Growth curve for T47D control and ZRF1 knockdown cells during 10 days. Data represent the average of three experiments, ± S.E.M. ^*^p < 0.5, calculated by two-tailed unpaired *t* test. (**G**) Cell death assay by trypan blue-positive cell count in control and ZRF1 depleted T47D cells during 10 days. Data represent the average of three experiments. (**H**) Flow cytometry analysis of cell cycle distribution of control and shZRF1 cells. Data represent the average of three experiments, ± S.E.M. ^*^p < 0.5, ^**^p < 0.01 calculated by two-tailed unpaired *t* test.

Breast cancer is a heterogenous disease in which each subtype displays different prognosis and requires different therapy options. Given the role of ZRF1 in other types of cancer, we first aimed to explore which type of breast cancer is mainly associated with ZRF1. We analyzed the DNA copy number and mRNA expression changes of ZRF1 employing the TCGA breast cancer dataset (dated June 30th, 2016) and by using the publicly available cBioPortal software [46, 47]. According to the alteration frequency analysis, most of the DNA copy changes (mutation, deletion or amplification) related to ZRF1 are enriched in breast invasive ductal carcinoma (Supplementary Figure 1A). Based on the mRNA expression changes of ZRF1, breast invasive ductal carcinoma is ranked first among other breast cancer types (96.4%) (Supplementary Figure 1B). So far ZRF1’s role in breast cancer was only studied in the MCF7 cell line, which represents an *in vitro* model of breast invasive ductal carcinoma [48]. In this study, we aimed to explore ZRF1’s role in breast cancer progression in a more elaborate way and performed experiments in both ER (+) (MCF7, T47D) and ER (–) (MDA-MB-231, MDA-MB-453) cell lines. After generating either MCF7 or T47D cells expressing a non-specific shRNA (Control) or shRNA targeting ZRF1 (shZRF1) by viral infection (Figure 1A and Supplementary Figure 13A; MCF7 cells, Figure 1E and Supplementary Figure 13B; T47D cells), we assessed the growth rates of both cell lines. After seeding the same number of cells, we counted cells during 10 days and generated a growth curve (Figure 1B; MCF7 cells, Figure 1F; T47D cells). Although ZRF1 knockdown MCF7 cells grew less compared to control cells, a significant difference in the growth rate was only observed at days 4 and 6. Similarly, ZRF1 knockdown T47D cells grew significantly less only at day 8. Next, we investigated if the observed decrease in cell growth was caused by an increase in apoptosis and/or a reduction in proliferation. Judged by trypan blue staining, we did not observe a significant difference in the live/dead cell ratio between control and ZRF1 depleted MCF7 or T47D cells under normal growth conditions (Figure 1C; MCF7 cells, Figure 1G; T47D cells). To quantify the actively dividing cells in S phase we performed BrdU staining and analyzed the cell cycle profile of both cell lines using flow cytometry. Whereas control MCF7 cells had an average of 33.2% cells in S phase and 14.62% cells in G2-M phase, ZRF1 knockdown cells had an average of 27.03% cells in S phase and 9.43% cells in G2-M phase (Figure 1D and Supplementary Figure 2A). On the other hand, knockdown of ZRF1 had only a slight effect on the cell cycle profile of T47D cells. Control T47D cells showed an average of 21.6% cells in S phase and 6.38% cells in G2-M phase. ZRF1 depleted cells had an average of 19.57% cells in S phase and 9.52% cells in G2-M phase (Figure 1H and Supplementary Figure 2B). Collectively these data suggest that knockdown of ZRF1 has a mild effect on the cell proliferation properties in MCF7 and T47D cells.

### Knockdown of ZRF1 facilitates the gain of metastatic features in MCF7 and T47D cells

**Figure 2:**
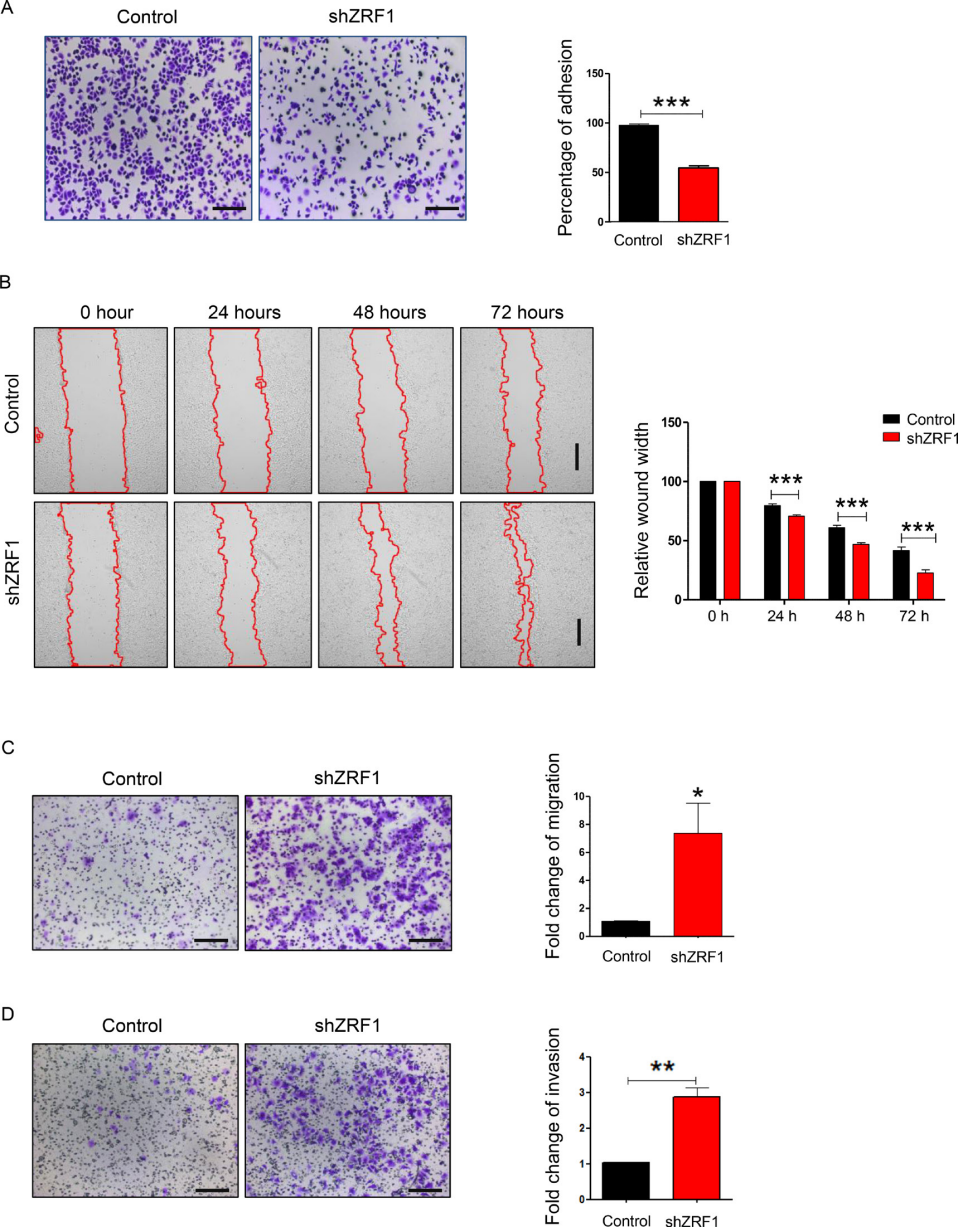
ZRF1 is a key player in the regulation of cellular adhesion, cell migration and cell invasion in MCF7 cells (**A**) Representative microscopy images of control and ZRF1 knockdown cells attached to collagen coated plates after 1 hour of incubation. Scale bar, 200 µm. Cell adhesion was calculated as fold change of crystal violet fluorescence. Data represent the average of three experiments, ± S.E.M. ^***^p < 0.001 as calculated by two-tailed unpaired *t* test. (**B**) Wound healing assay of control and shZRF1 cells during 72 hours. Images were taken every 24 hours. Scale bar, 500 µm. Quantification of wound healing was calculated as relative wound width after normalization of each value in relation to the 0 time point of each cell line. Data represent the average of three experiments, ± S.E.M. ^***^p < 0.001 as calculated by two-tailed unpaired *t* test. (**C**) Representative microscopy images of transwell migration assays of control and shZRF1 cells during 24 hours. Scale bar, 200 µm. Quantification of the transwell cell migration assay: Migrated cells from 12 different wells of a 96-well transwell plate were counted and calculated as fold change. Data represent the average of three experiments, ± S.E.M. ^**^p < 0.01, calculated by two-tailed unpaired *t* test. (**D**) Representative microscopy images of Matrigel coated transwell invasion assays of control and shZRF1 cells after 72 hours. Scale bar, 200 µm. Quantification of the cell invasion assay: Invaded cells from 12 different wells of 96-well transwell plate were counted and calculated as fold change. Data represent the average of three experiments, ± S.E.M. ^**^p < 0.01, calculated by two-tailed unpaired *t* test.

**Figure 3:**
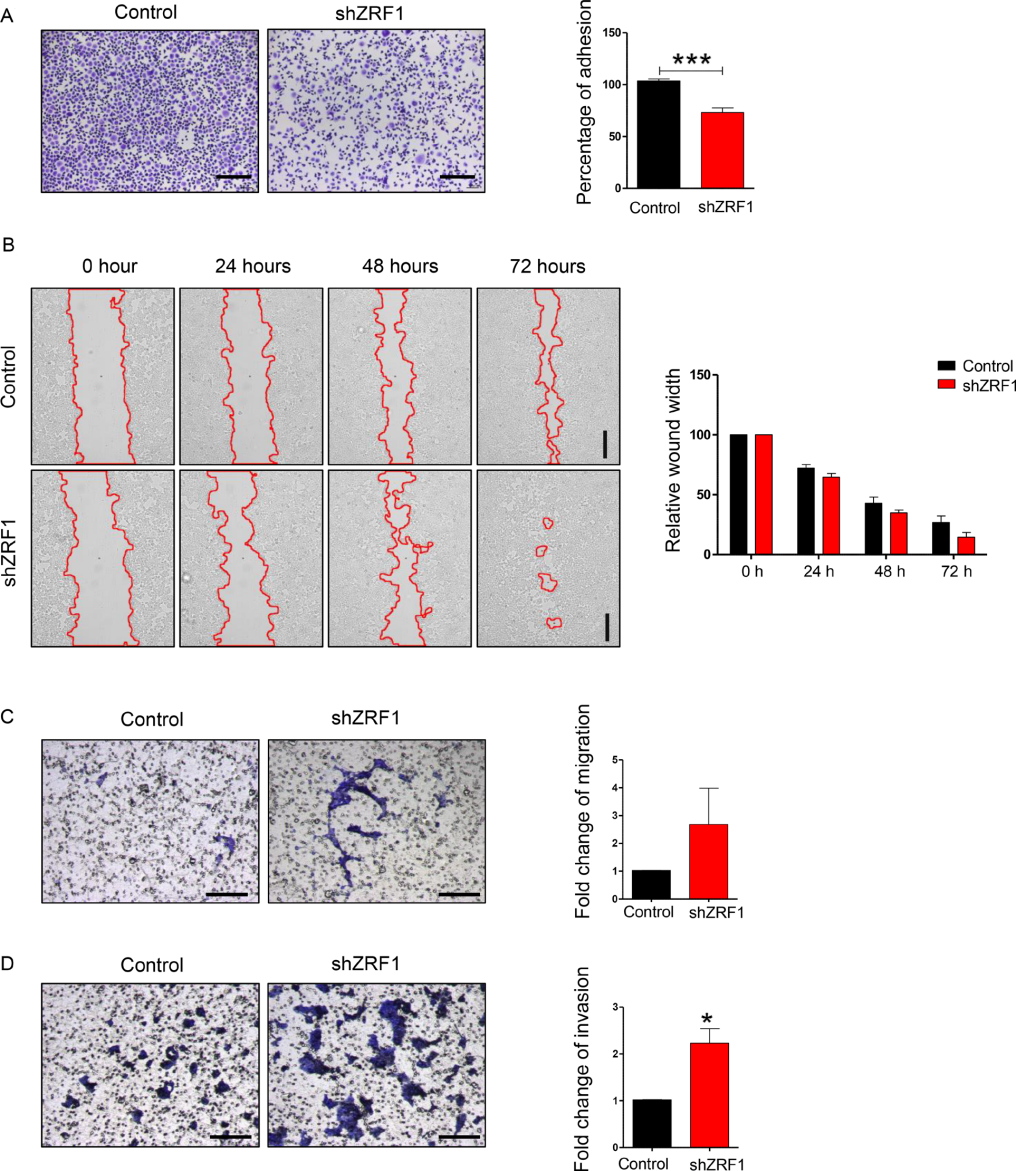
ZRF1 is a key player in the regulation of cellular adhesion, cell migration and cell invasion in T47D cells (**A**) Representative microscopy images of control and ZRF1 knockdown cells attached to collagen coated plates after 3 hours of incubation. Scale bar, 200 µm. Cell adhesion was calculated as fold change of crystal violet fluorescence. Data represent the average of three experiments, ± S.E.M. ^***^p < 0.001 as calculated by two-tailed unpaired *t* test. (**B**) Wound healing assay of control and shZRF1 cells during 72 hours. Images were taken every 24 hours. Scale bar, 500 µm. Quantification of wound healing was calculated as relative wound width after normalization of each value in relation to the 0 time point of each cell line. Data represent the average of three experiments. (**C**) Representative microscopy images of transwell migration assays of control and shZRF1 cells during 48 hours. Scale bar, 200 µm. Quantification of the transwell cell migration assay: Migrated cells from 3 different transwell inserts of a 24-well plate were counted and calculated as fold change. Data represent the average of three experiments. (**D**) Representative microscopy images of Matrigel coated transwell invasion assays of control and shZRF1 cells after 72 hours. Scale bar, 200 µm. Quantification of the cell invasion assay: Invaded cells from 3 different transwell inserts of a 24-well plate were counted and calculated as fold change. Data represent the average of three experiments, ± S.E.M. ^*^p < 0.5, calculated by two-tailed unpaired *t* test.

Next we examined shZRF1 and control cell lines microscopically to obtain further insight into ZRF1 function in ductal invasive breast carcinoma. For both, the MCF7 and T47D cell lines, we observed that knockdown of ZRF1 exhibited an altered cellular morphology when compared to control cells (Supplementary Figure 3A; MCF7 cells, Supplementary Figure 3C; T47D cells). Whereas control cells grew in cell clusters and showed a typical cobblestone-like phenotype, shZRF1 cells did not form cell clusters and demonstrated a spindle-like, elongated phenotype. Phalloidin staining in the MCF7 cell line further revealed abnormal actin structures and decreased levels of cell-cell interaction after knockdown of ZRF1 (Supplementary Figure 3B). Control MCF7 cells displayed intensely stained actin fibers which are tightly connected. In contrast, ZRF1 knockdown MCF7 cells showed poorly stained actin fibers and distorted cell connections. One of the crucial features of metastatic cancers is their tendency to lose cell adhesion [49]. Thus, our observations prompted us to evaluate the adhesive capacity of both cell lines in more detail. For this purpose, we performed cell adhesion assays using collagen-coated cell culture plates. After incubating these plates with either MCF7 or T47D cell lines, we stained the cells retained on the collagen matrix with crystal violet and measured its absorbance (Figure 2A; MCF7 cells, Figure 3A; T47D cells). Compared to control cells, we noticed almost 50% less crystal violet fluorescence in ZRF1 depleted MCF7 cells (Figure 2A) and almost 30% less crystal violet in ZRF1 depleted T47D cells (Figure 3A). These results suggest a significant decrease in cellular adhesion upon knockdown of ZRF1 *in vitro*. Tumor cells often exhibit a decrease in cell–cell and/or cell–matrix adhesion which is accompanied by increased cell motility. To explore whether ZRF1 depletion also leads to increased cell motility, we carried out both, wound healing and migration assays. To this end we generated a scar tissue in the monolayer of control and shZRF1 cells. Subsequently, we monitored the wound healing capacity during 72 hours and captured images every 24 hours. ZRF1 knockdown MCF7 cells closed their wound significantly faster than control cells at all time points analyzed (Figure 2B). Detailed microscopy images of the wound area revealed that shZRF1 cells moved in single cells and preserved their spindle-like morphology whereas control cells moved rather in cell clusters and exhibited tight cell-cell connections during the wound healing process (Supplementary Figure 4A and 4B). ZRF1 knockdown T47D cells exhibited a similar behavior and closed their wound faster than control cells (Figure 3B). To further support the observed increased cell motility in shZRF1 cells, we assessed the migratory potential of both cell lines using a transwell system. In this system, control and shZRF1 cells were resuspended in serum free medium and added into the upper chamber of the transwell system. The lower chamber was filled with complete medium supplemented with 20% FBS serving as a chemoattractant. Whereas ZRF1 knockdown MCF7 cells migrated almost 7.3 fold more compared to control cells within 24 hours, ZRF1 knockdown T47D cells migrated almost 2.6 fold more compared to control cells within 48 hours (Figure 2C; MCF7 cells, Figure 3C; T47D cells). Taken together, ZRF1 likely limits the migration of cancerous cells into other tissues *in vitro*. Reduction in cell adhesion and increased cell migration is usually correlated with tumor invasion and metastasis. Therefore, we next assessed the invasive capacity of ZRF1 knockdown cells addressing one of the critical steps in tumor metastasis. Cell invasion of both cell lines was determined by measuring cell invasion through a Matrigel barrier in a transwell system. Control and shZRF1 cells were plated in serum free medium on top of the Matrigel-coated inserts, whereas the bottom wells contained complete medium with 20% FBS. After 72 hours we counted the invaded cells in the lower chamber. We noticed that shZRF1 MCF7 cells invaded into the serum containing medium almost 3 fold more and shZRF1 T47D cells invaded 2.2 fold more compared to their control cells (Figure 2D; MCF7 cells, Figure 3D; T47D cells). Collectively, knockdown of ZRF1 promotes the development of metastatic properties in MCF7 and T47D cell lines, which may result in a more aggressive breast cancer phenotype.

### ZRF1 depletion provokes an aggressive cancer phenotype in a 3D environment

**Figure 4:**
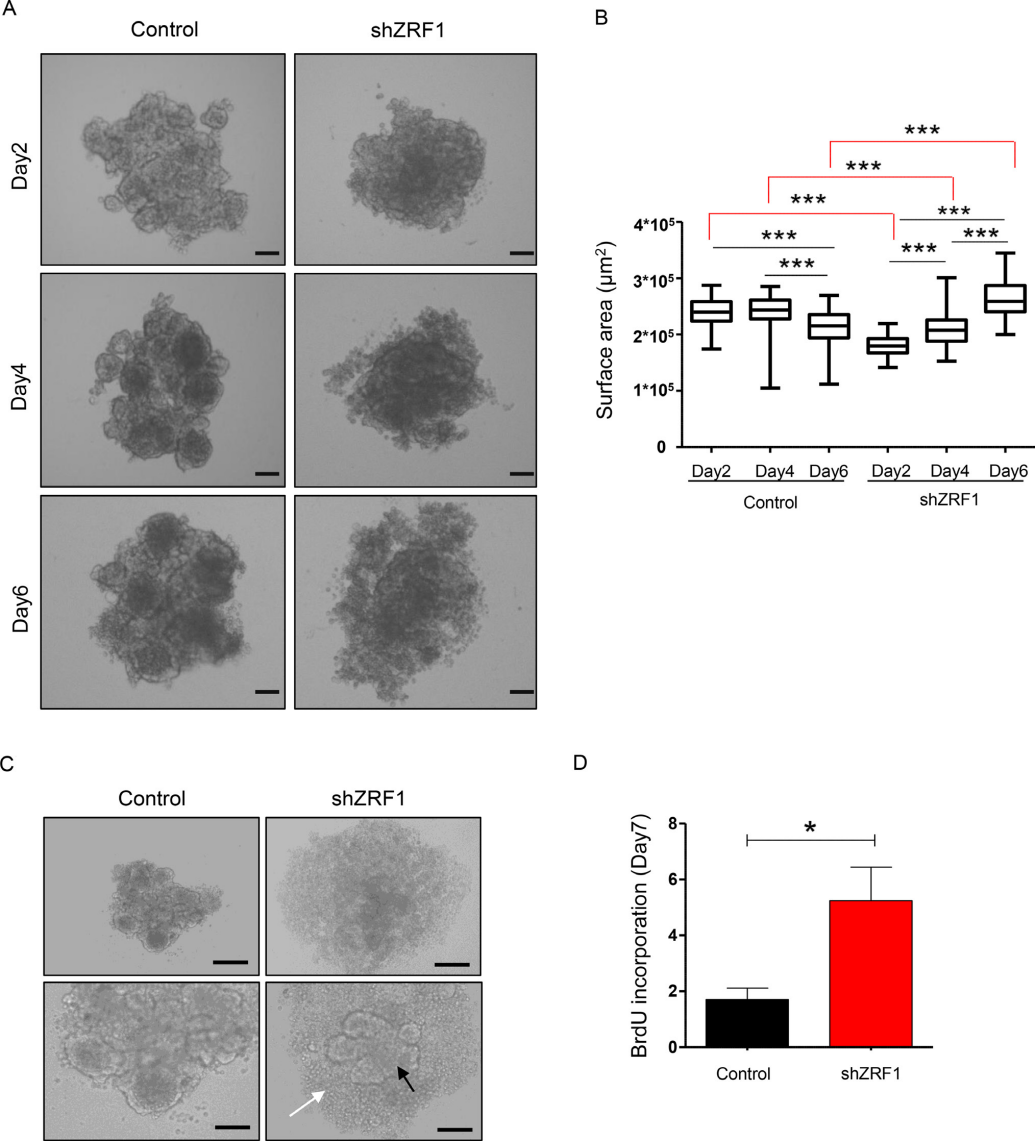
ZRF1 depleted MCF7 cells exhibit a loose phenotype and increase the cell proliferation rate in 3D culture (**A**) Brightfield images of the spheroids derived from control and shZRF1 cells were taken at 10× magnification at days 2, 4 and 6. Scale bars, 200 µm. (**B**) Quantification of the surface area of the spheroids derived from control and ZRF1 knockdown cells during 6 days. The surface area of the each spheroid was calculated using the ImageJ software. The whiskers of the plots represent the minimum and maximum values of the population of counted spheroids. ^***^p < 0.001 as calculated by two-tailed unpaired *t* test. (**C**) Representative microscopy images of control and shZRF1 MCF7 cells after 7 days in suspension culture. Whereas control cells generated mass type spheroids with disorganized nuclei and tight cell-cell junctions, shZRF1 cells generated smaller mass type of spheroids with decreased cell-cell adhesion. A significantly increased amount of single cells (white arrow) outside of the original solid mass (black arrow) was also observed in the spheroids derived from ZRF1 knockdown cells. Scale bars, upper panel 200 µm, lower panel 100 µm. (**D**) BrdU cell proliferation assay in control and ZRF1 depleted cells. The incorporated BrdU levels in 7 days old spheroids derived from control and shZRF1 were calculated. Data represent the average of three experiments, ± S.E.M. ^*^p < 0.1, calculated by two-tailed unpaired *t* test.

Having shown that ZRF1 depletion causes the acquisition of metastatic features in 2D culture, we next addressed if the increased metastatic capacity imposes an aggressive cancer phenotype in 3D culture. In 3D culture cancer cells are forced to grow in an anchorage-independent manner leading to the establishment of well-rounded spheroids, which provide valuable information about the cell behavior of epithelial tissues within a microenvironment as observed *in vivo* [50]. Hence, we set out to study ZRF1 function in a 3D culture system employing MCF7 cells since they already exhibited a more dramatic phenotype upon ZRF1 knockdown in 2D culture. After coating 96-well plates with agarose to prevent cell attachment and to force cell growth, we monitored the epithelial morphology of control and ZRF1 depleted cells during 7 days (Figure 4A). In suspension culture, control cells generated “a mass type” of spheroids consisting of cells with disorganized nuclei and tight cell-cell junctions. Furthermore, these spheroids became packed and decreased their surface area over time (Figure 4B). Initially, shZRF1 cells generated a smaller “a mass type” of spheroids with decreased cell-cell adhesion, however, they became larger and increased their surface area over time (Figure 4A and 4B). A significantly increased amount of single cells outside of the original solid mass was also observed in the spheroids derived from ZRF1 knockdown cells (Figure 4C). To clarify whether the observed increase in the cell surface area was not just an artefact of decreased cell adhesion but also a result of increased cell proliferation, we treated 7 days old spheroids with BrdU and dissociated them into single cells. Analysis of BrdU incorporation of these single cells by flow cytometry revealed that single cells within the spheroids derived from shZRF1 cells incorporated almost 3.5 fold more BrdU compared to control cells (Figure 4D and Supplementary Figure 5A). Taken together, ZRF1 depletion in MCF7 cells causes a loose spheroid phenotype *in vitro* as a result of decreased cell adhesion and increased cell proliferation.

**Figure 5:**
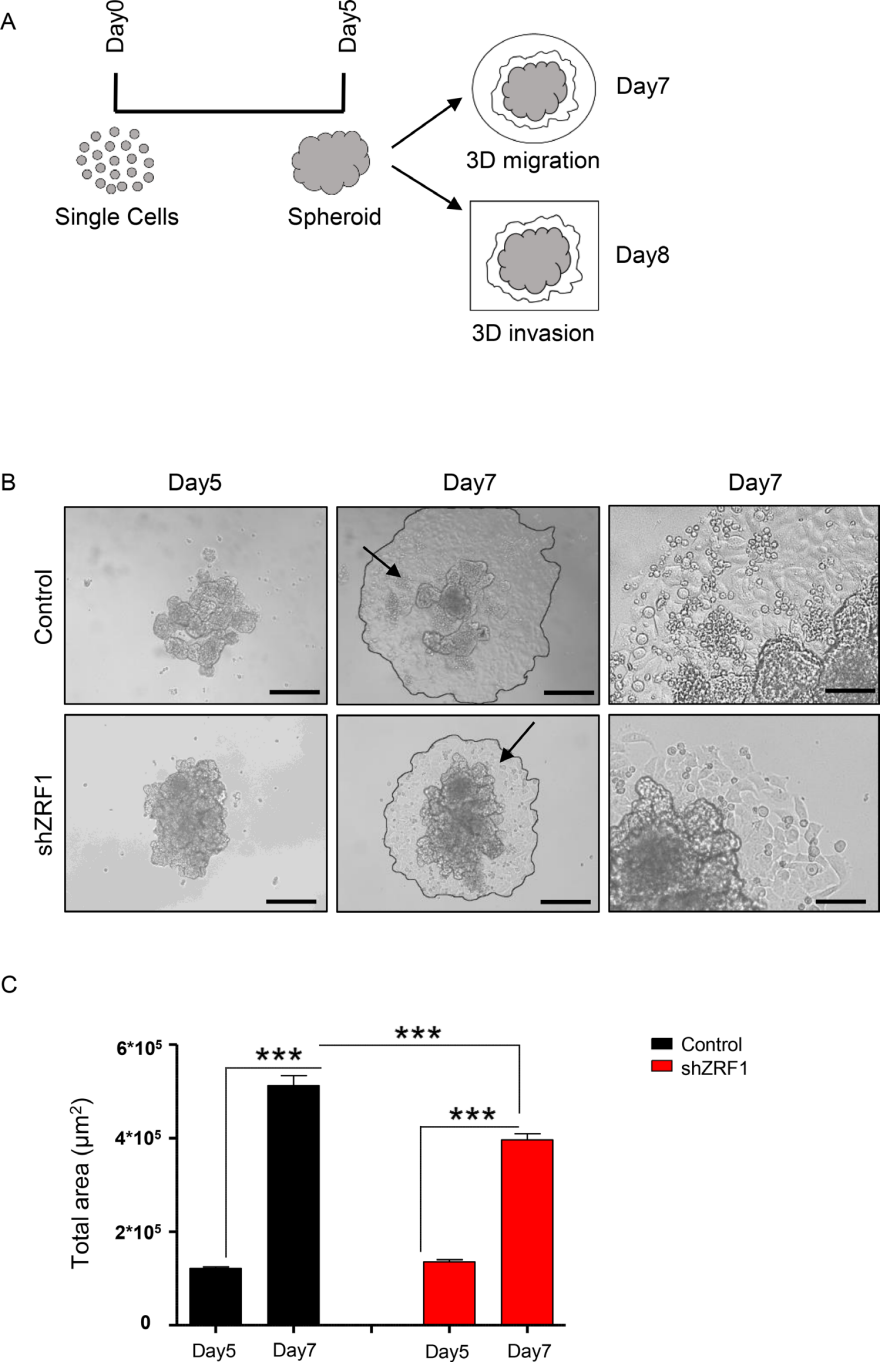
Spheroids derived from control MCF7 cells exhibit more migration than spheroids derived from shZRF1 MCF7 cells in a 3D environment (**A**) Representative scheme of the experimental procedure employed during 3D migration and invasion assays. After 5 days of suspension culture in low attachment 96-well plates, spheroid structures were generated from single cells in a 3D environment. Half of the spheroids were transferred into gelatin coated 96-well plates and were cultured until day 7 to measure the migration area. The other half of the spheroids were embedded in Matrigel in µ-Slide 8 Well chambers and were cultured until day 8 to measure the invasion area. (**B**) Brightfield images of the spheroids derived from control and shZRF1 cells were taken at 10× magnification at days 5 and 7. Black arrows indicate the magnified area of day7 images. Scale bars, 200 µm, 100 µm. (**C**) 3D migration was calculated as difference between the total area of spheroids at day 5 and the total area of the spheroids at day 7. Data represent the average of three experiments, ± S.E.M. ^***^p < 0.001 as calculated by two-tailed unpaired *t* test.

**Figure 6:**
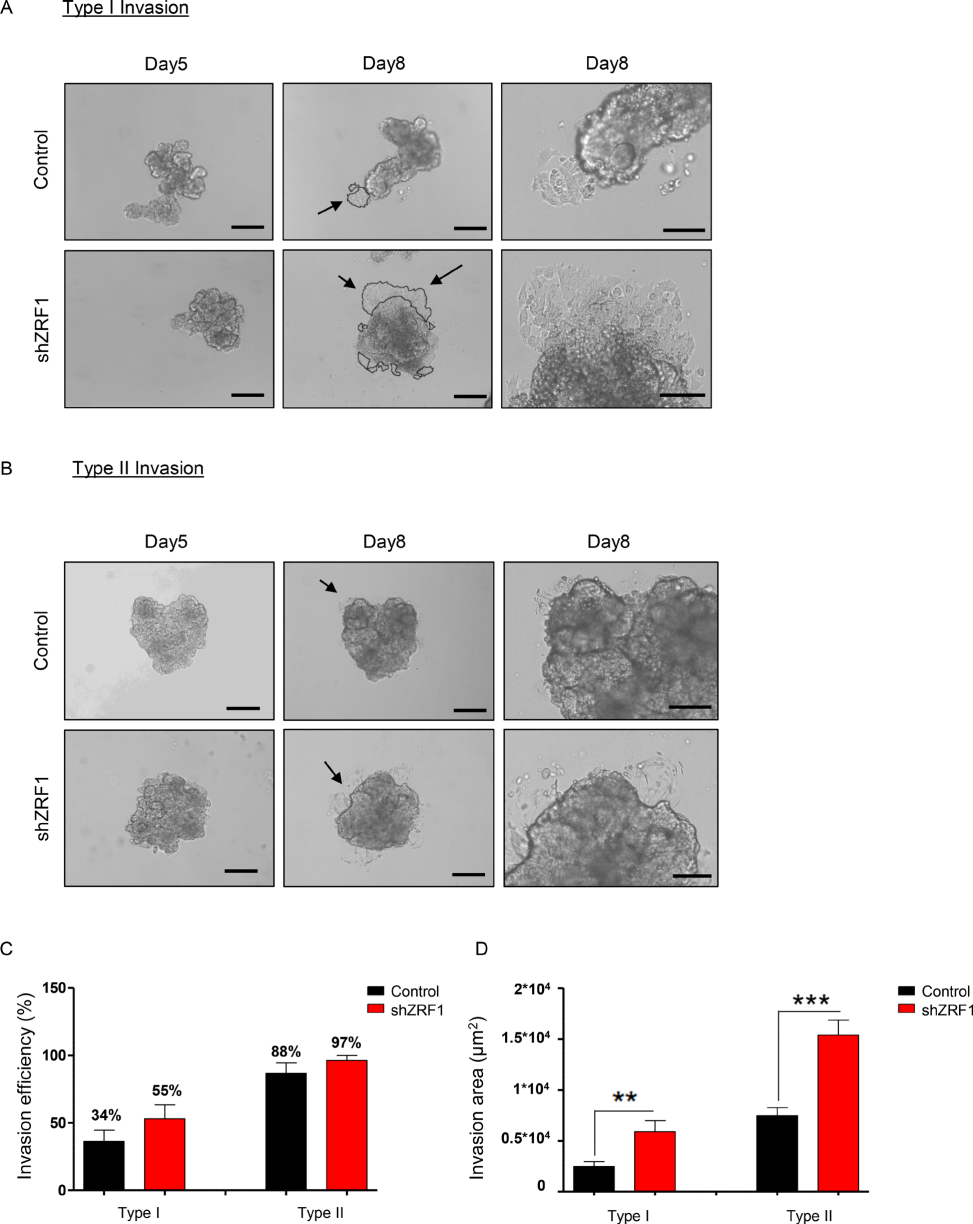
Spheroids derived from ZRF1 depleted MCF7 cells exhibit more invasion in a 3D environment (**A**) Type I Invasion: Brightfield images of the spheroids derived from control and shZRF1 cells were taken at 10× magnification at days 5 and 8. Black arrows indicate the magnified area of day8 images. Scale bars, 200 µm, 100 µm. (**B**) Type II Invasion: Brightfield images of the spheroids derived from control and shZRF1 cells were taken at 10× magnification at days 5 and 8. Black arrows indicate the magnified area of day8 images. Scale bars, 200 µm, 100 µm. (**C**) Invasion efficiency of each cell line was calculated as percentage of the spheroids which display either Type I or Type II invasion. (**D**) 3D invasion was calculated as invasion area in 8 days old spheroids derived from control and shZRF1 cells based on whether they exhibit Type I or Type II invasion.

We next performed three-dimensional functional assays to explore the metastatic capacity of the spheroids derived from control and shZRF1 cells (Figure 5A). To examine 3D migration, we utilized a tumor-spheroid based migration assay that resembles tumor cell dissemination from a solid microtumor or micrometastases [51]. After generating 3D spheroids in 96-well ultra-low attachment plates, we transferred 5 days old spheroids into gelatin coated 96-well plates and allowed them to disseminate into the coated surface (Figure 5B). Although both control and ZRF1 knockdown cells exhibited 100% migration efficiency, control cells occupied a significantly larger area compared to shZRF1 cells after 48 hours (Figure 5C). Next, to explore 3D invasion, we embedded 5 days old spheroids into growth factor reduced Matrigel and analyzed the invasion capacity for 72 hours. Matrigel here served as a celular matrix, which cells first need to degrade proteolytically in order to migrate into the surrounding tissue. We classified the observed cell invasions into 2 different types. In Type I invasion, cells formed extended invadopodia to migrate into the surrounding tissue reflecting a particularly aggressive invasive behavior (Figure 6A). In Type II invasion, cells exhibited a rather limited invasion with cells dispersing from the original spheroid core showing a mixed morphology of stellate and single cells (Figure 6B). Notably, shZRF1 cells displayed a higher invasion potential on Matrigel particularly for the type I invasion (Figure 6C). Moreover, shZRF1 cells significantly invaded into a much larger area than control cells for both types of invasion (Figure 6D).

Given the loose spheroid phenotype and increased metastatic capacity observed in shZRF1 cells, we next asked if this phenotype is rooted in the epithelial-mesenchymal-transition (EMT). During EMT epithelial cells lose their junctions and apical–basal polarity, change their signaling and gene expression programs [52, 53], which lead to increased cell motility and an invasive phenotype. To address a role for ZRF1 in EMT, we collected spheroids from both cell lines after 7 days of culture, dissociated them into single cells and performed RT-qPCR experiments to monitor EMT related genes including cell surface markers, cytoskeletal marker, extracellular matrix (ECM) and transcription factors (Supplementary Figure 5B). We observed that all the transcription factors tested and Fibronectin, which is an extracellular matrix component, were upregulated in ZRF1 knockdown cells. In contrast, the expression levels of E-cadherin, N-cadherin and Vimentin remained unchanged upon ZRF1 knockdown. Immunostaining images of E-cadherin in 7 days old spheroids confirmed the previous PCR data regarding the E-cadherin expression (Supplementary Figure 5C). As the E cadherin to N cadherin transition did not occur despite upregulation of almost all transcription factors tested, we decided to study a potential role of ZRF1 in EMT in an additional cell line. To this end, we used the MDA-MB-231 cell line, which is representative of triple negative breast invasive ductal cancers and which is devoid of ER (estrogen receptor), PR (progesterone receptor) and HER2 (human epidermal growth receptor) [48, 54]. After generating MDA-MB-231 cells either expressing a non-specific shRNA (Control) or shRNA targeting ZRF1 (shZRF1) (Supplementary Figure 6A), we generated 3D spheroids and monitored them during 7 days (Supplementary Figure 6B). Similar to MCF7 cells, ZRF1 depleted MDA-MB-231 cells displayed a loose spheroid phenotype compared to control cells, particularly visible at day 2. Even though spheroids derived from both cell lines became packed over time, the surface area of the spheroids derived from shZRF1 cells was bigger than the surface area of spheroids derived from control cells at all time points (Supplementary Figure 6C). Furthermore, an increased amount of single cells around the original cell mass was also observed when analyzing spheroids derived from shZRF1 cells, particularly visible at day 6 (Supplementary Figure 6B). Next we performed RT-qPCR analysis of EMT related genes in single cells dissociated from 7 days old spheroids derived from control and ZRF1 knockdown MDA-MB-231 cells. Whereas most of the genes tested remained unchanged upon ZRF1 depletion, only SNAIL1 was significantly upregulated in shZRF1 cells (Supplementary Figure 6D). Taken together the 3D phenotype exhibited by shZRF1 cells seems most likely to be a consequence of ZRF1’s involvement in other cellular pathways and/or signaling mechanisms rather than EMT related pathways. Nevertheless, knockdown of ZRF1 provokes an aggressive cancer phenotype in suspension culture and spheroids derived from shZRF1 cells possess high potential for excessive tumor growth and metastasis.

### Transcription programs facilitating an aggressive breast cancer phenotype are controlled by ZRF1

**Figure 7:**
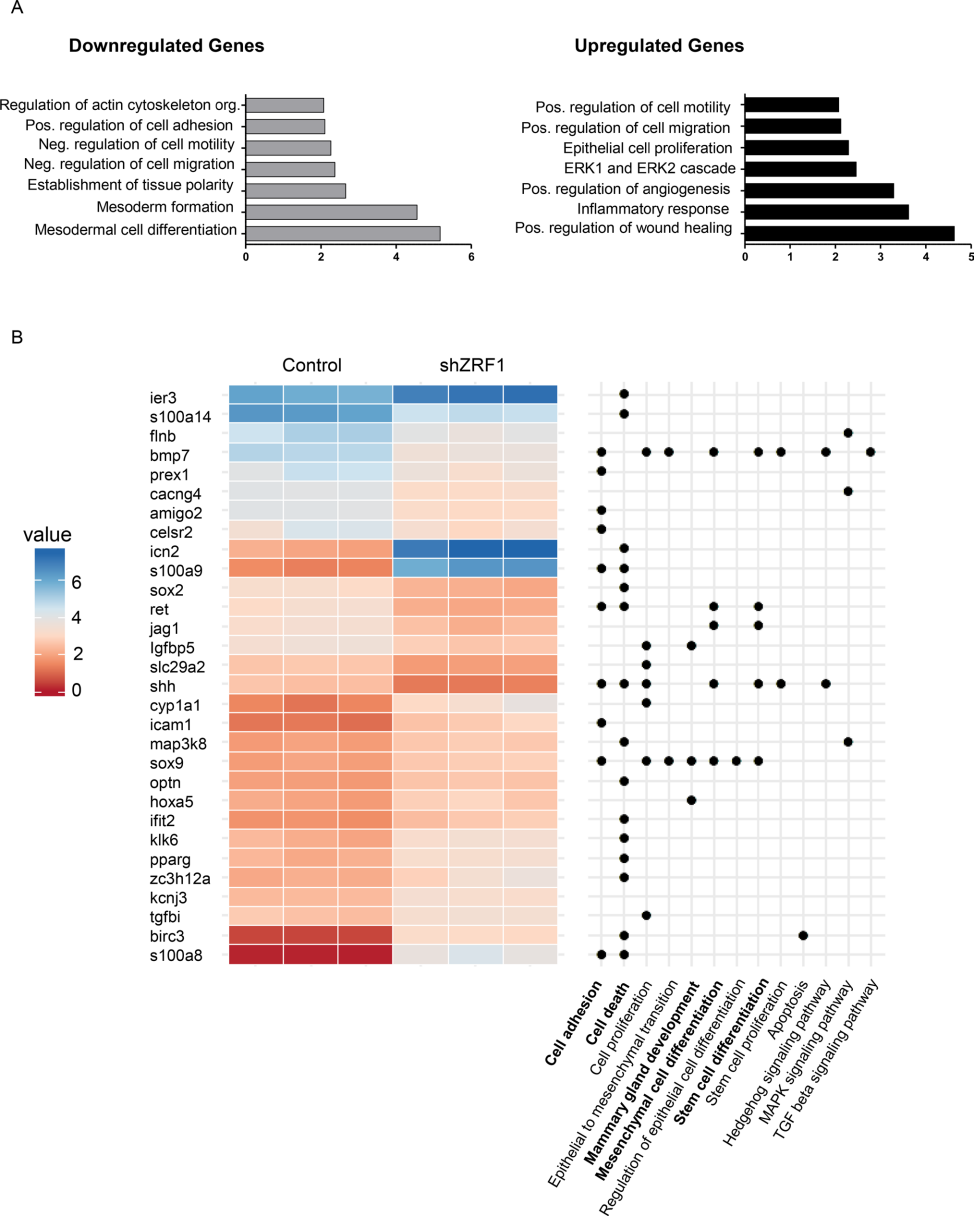
Transcription programs facilitating an aggressive breast cancer phenotype are controlled by ZRF1 (**A**) Gene Ontology (GO) biological processes enriched for down- and upregulated genes upon ZRF1 depletion in MCF7 cells. Enrichment (x-axis) is presented as the fold change of the observed/expected ratio. (**B**) Expression levels of differentially expressed genes related to GO terms and KEGG pathways of interest.

In order to gain more insight into how ZRF1 is involved in the progression of breast invasive ductal carcinoma, we carried out RNA-sequencing of 7 days old spheroids derived from control and shZRF1 MCF7 cells grown in a 3D environment. After quantifying the gene expression levels from both cell lines, we performed differential expression analysis followed by gene set enrichment analysis with the genes that significantly changed between conditions (FDR < 0.05, and expression fold change of at least 2-fold between conditions). According to the Gene Ontology (GO) analysis, genes that are upregulated upon ZRF1 knockdown are highly enriched in biological processes including positive regulation of wound healing, inflammatory response, positive regulation of angiogenesis, the ERK1/2 cascades, epithelial cell proliferation and positive regulation of cell migration and motility (Figure 7A). Genes that are downregulated upon ZRF1 knockdown are highly enriched in biological processes including mesodermal cell differentiation, mesoderm formation, establishment of tissue polarity, negative regulation of cell migration and motility, positive regulation of cell adhesion and regulation of actin cytoskeleton organization likely reflecting the observed phenotype of shZRF1 MCF7 cells. To better illustrate the most differentially expressed genes (Supplementary Figure 7A), we clustered differently expressed genes [FDR < 0.05, at least 80% changes in expression between conditions and a moderate expression in at least one replicate (log_2_ RPKM > 5)] according to their expression levels and with respect to GO biological processes or KEGG pathways involved in breast cancer progression (Figure 7B). The largest group of genes significantly regulated by ZRF1 is related to either cell death (15/116 genes, *p*-value 0.002) or cell adhesion (10/116 genes, *p*-value 0.23). Genes essential for mesenchymal or stem cell differentiation and mammary gland development represent the second largest group of genes (total 14/116 genes, *p*-value 0.08) affected by ZRF1 depletion which is in keeping with the regulatory role of ZRF1 during development [35]. To investigate whether the differentiation potential of ZRF1 knockdown MCF7 cells is impaired, we conducted cell differentiation experiments as published before [55]. We treated both control and shZRF1 cell lines with retinoic acid (RA) for 4 days to induce adipose tissue differentiation. Following the determination of adipose tissue by Nile Red staining (Supplementary Figure 7B), we counted the lipid droplets in control and shZRF1 cells (Supplementary Figure 7C). As microscopy images indicated, control cells were responsive to RA mediated differentiation and were capable of generating 2 times more lipid droplets after the treatment. In contrast shZRF1 MCF7 cells were not responsive to RA mediated differentiation and failed to generate lipid droplets. Interestingly, shZRF1 cells had more lipid droplets in vehicle treated control conditions compared to the RA treated conditions suggesting an abnormal differentiation capacity (Supplementary Figure 7C). In addition, the transcriptome data indicates a central role for ZRF1 in the regulation of cell movement in MCF7 cells (Figure 7A). To identify if this finding is not just limited to ER (+) breast ductal carcinoma (MCF7), we additionally performed wound healing assays in ER (–) breast ductal carcinoma cell lines (MDA-MB-231, MDA-MB-453). After generating control and shZRF1 cell lines in MDA-MB-453 cells by viral infection (Supplementary Figure 9A), we monitored the wound healing capacity of shZRF1 cells either during 24 hours for the MDA-MB-231 cells (Supplementary Figure 8B and 8C) or 72 hours for the MDA-MB-453 cells (Supplementary Figure 9B and 9C). However, we did not observe a significant difference in the wound healing capacity comparing control and ZRF1 knockdown conditions in both cell lines. Unlike for MCF7 cells (data not shown), we were able to overexpress ZRF1 in MDA-MB-231 cells (Supplementary Figures 8A and 13C). Surprisingly, overexpression of ZRF1 decreased the wound healing capacity of MDA-MB-231 cells dramatically (Supplementary Figure 8B and 8C). Flow cytometry analysis of spontaneous cell death (Supplementary Figure 10A) and cell cycle (Supplementary Figure 10B) in ZRF1 overexpressing MDA-MB-231 cells confirmed that decreased wound healing capacity was a direct effect of ZRF1 on cell motility. In sum, our genomic data reveals likely a central role for ZRF1 in regulating almost all the critical steps in the metastatic cascade including cell polarity, cell adhesion, cell migration, cell differentiation and cell death.

### In the absence of ZRF1, breast cancer cells become less responsive to both anti-hormonal therapy and chemotherapy

**Figure 8:**
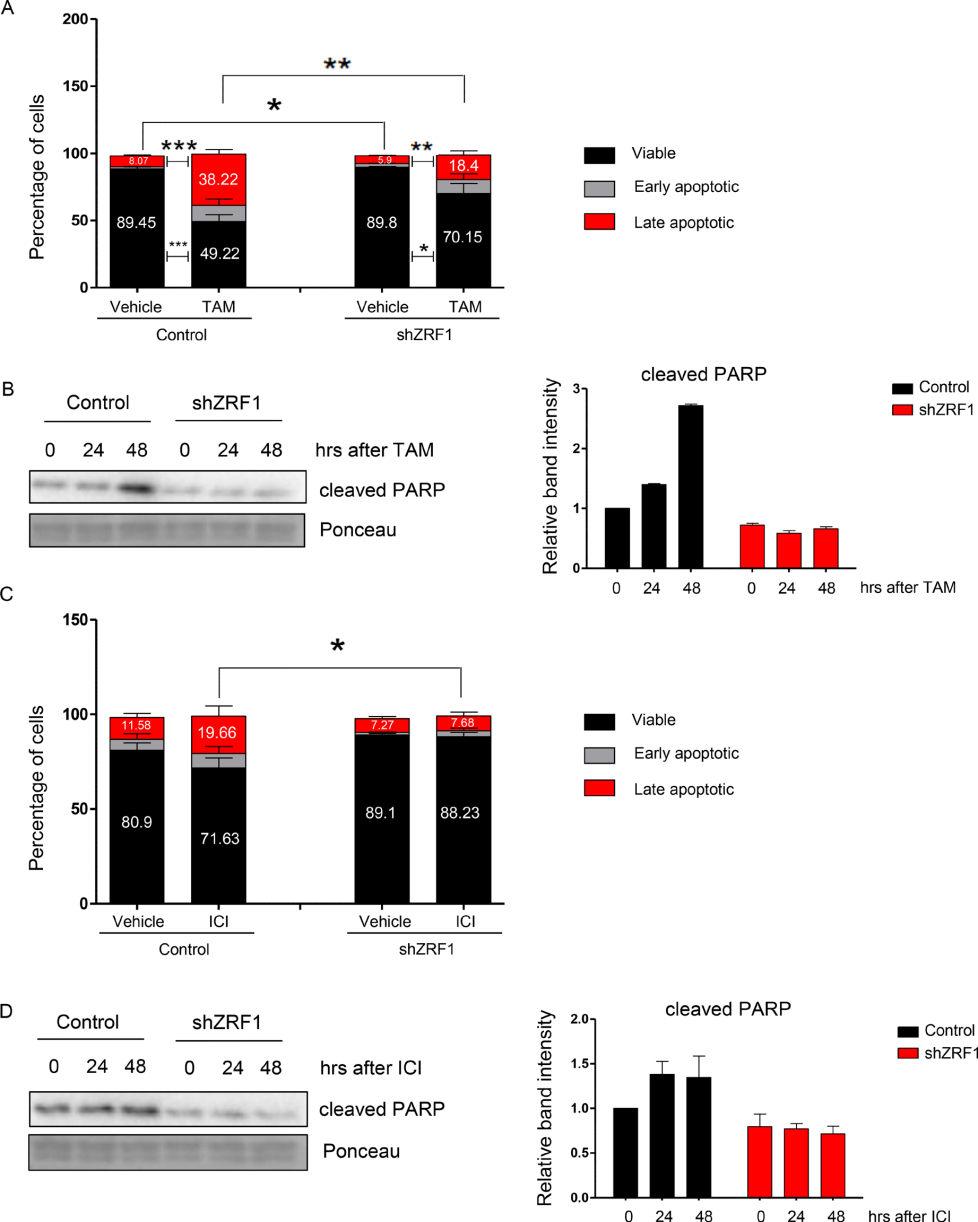
ZRF1 depletion provokes resistance to cell death in MCF7 cells (**A**) Flow cytometry analysis of apoptotic cell distribution after 48 hours of 10 μM Tamoxifen treatment in control and shZRF1 cells. Data represent the average of three experiments, ± S.E.M. ^*^p < 0.5, ^**^p < 0.01, ^***^p < 0.001 calculated by two-tailed unpaired *t* test. (**B**) Western blot of cleaved PARP protein in control and shZRF1 cells after a time course of Tamoxifen treatment. Ponceau was used as a loading control. Relative band intensity of each sample was calculated in relation to the control (0 hour band) intensity: (Relative Band Intensity of Cleaved PARP/ Relative Band Intensity of Ponceau). (**C**) Flow cytometry analysis of apoptotic cell distribution after 7 days of 100 nM ICI 182,780 treatment in control and shZRF1 cells. Data represent the average of three experiments, ± S.E.M. ^*^p < 0.5, calculated by two-tailed unpaired *t* test. (**D**) Western blot of cleaved PARP protein in control and shZRF1 cells after a time course of 1 μM ICI 182,780 treatment. Ponceau was used as a loading control. The relative band intensity of each sample was calculated according to the control sample (0 hour band) intensity: (Relative Band Intensity of Cleaved PARP/ Relative Band Intensity of Ponceau).

Based on the transcriptome data a large number of cell death related genes is controlled by ZRF1. Thus, we decided to investigate the cell death response in ZRF1 knockdown cells in more detail. To this end, we treated control and shZRF1 MCF7 cells with 10 µM 4-OH Tamoxifen (TAM) for 2 days. We used the same volume of vehicle as control for each experiment and synchronized cells for 48 hours prior to drug treatment. We analyzed the apoptotic response of both cell lines by flow cytometry based on their Annexin V and PI (Propidium Iodide) double staining (Figure 8A and Supplementary Figure 11A). Whereas control cells became dramatically apoptotic upon TAM administration, shZRF1 cells did not respond well to the treatment. Control cells increased the number of apoptotic cells (early + late apoptotic) 4.8 fold more compared to the control condition and had a total of 50.78% apoptotic cells after TAM treatment. On the contrary, shZRF1 cells increased the number of apoptotic cells (early + late apoptotic) only 2.9 fold more compared to their control condition and had a total of 29.85% apoptotic cells (early + late apoptotic) after TAM treatment. Western blot analysis of cleaved PARP protein, which is used as a hallmark of apoptosis, confirmed the decreased level of apoptosis in shZRF1 cells after TAM treatment (Figure 8B and Supplementary Figure 13D). Since ZRF1 knockdown MCF7 cells exhibited such a robust phenotype after TAM treatment, we investigated their response towards pure anti-estrogen ICI 182,780 (ICI), which is used for the treatment of Tamoxifen resistant breast cancer tumors in the clinic. After 48 hours of synchronization, we treated cells with 100 nM ICI 182,780 (ICI) during 7 days and analyzed their apoptotic response by flow cytometry (Figure 8C and Supplementary Figure 11B). In accordance with the previous results, control cells were responsive to ICI treatment. Upon drug administration, control cells increased the number of apoptotic cells (early + late apoptotic) 1.5 fold more compared to the control condition and had a total of 28.37% apoptotic cells. However, shZRF1 cells showed almost no response to ICI treatment and their total number of apoptotic cells remained unchanged. Western blot analysis of cleaved PARP further confirmed the resistance of ZRF1 knockdown cells towards ICI treatment (Figure 8D and Supplementary Figure 14A).

We next addressed if ZRF1 depletion promotes resistance to cell death in triple negative breast cancers (TNBC) which are biologically more aggressive than ER (+) breast cancers and are responsive to chemotherapeutic reagents such as platinum compounds [56]. Employing control and shZRF1 MDA-MB-231 cells, we performed MTT assays with different dosages of cisplatin to determine the optimal concentration of the drug (Supplementary Figure 12A) and decided to use a concentration of 100 µM cisplatin for further experiments. After treating control and shZRF1 cells with cisplatin for 24 hours, we analyzed the apoptotic response of control and shZRF1 cells by flow cytometry (Supplementary Figure 12B and 12C). Whereas control cells had a total of 24.3% apoptotic cells after cisplatin treatment, shZRF1 cells had only 5.4% apoptotic cells after the same treatment. Western blot analysis of cleaved PARP also revealed a dramatic difference between control and ZRF1 knockdown cells when utilizing two different dosages of cisplatin (Supplementary Figures 12D and 14B). Collectively these data indicate that ZRF1 is essential for the apoptotic response against anti-hormonal therapy and chemotherapy in breast cancer cells.

### ZRF1 depletion favors cell survival in both serum starved and drug treated conditions in MCF7 cells

**Figure 9:**
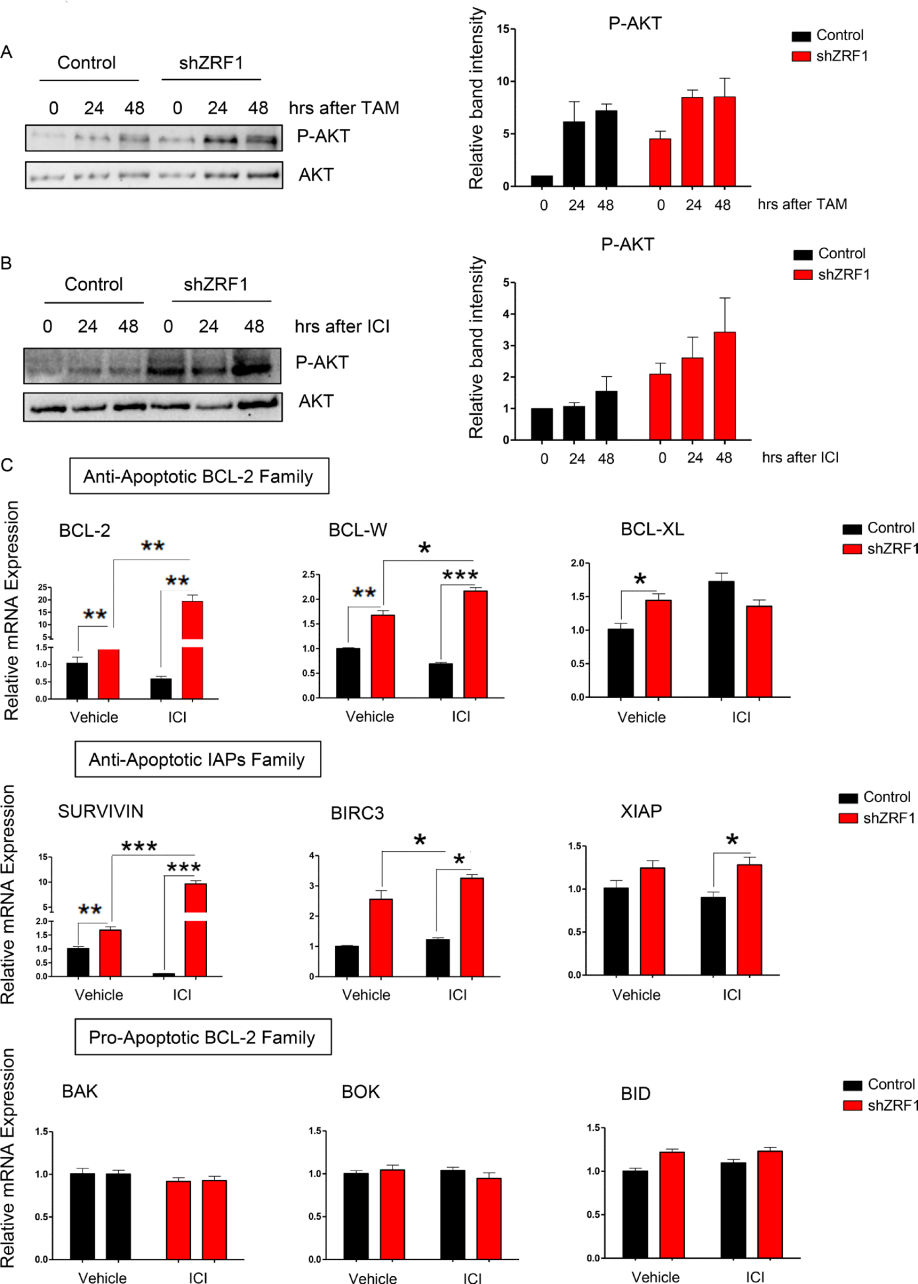
ZRF1 depletion favors cell survival in both serum deprived and anti-estrogenic drug treated conditions in MCF7 cells (**A**) Western blot of P-AKT and AKT proteins in control and shZRF1 cells after a time course of 10 μM Tamoxifen treatment. The active form of AKT protein was calculated as ratio of phosphorylated AKT levels (P-AKT) to total AKT levels in each condition. (**B**) Western blot of P-AKT and AKT proteins in control and shZRF1 cells after a time course of 1 μM ICI 182,780 treatment. The active form of AKT protein was calculated as ratio of phosphorylated AKT levels (P-AKT) to total AKT levels in each condition. (**C**) Real-time qPCR of BCL-2 and IAP family genes in control and shZRF1 cells after 1 μM ICI 182,780 treatment for 48 hours. Expression levels were normalized to the housekeeping gene GAPDH. Data represent the average of three experiments, ± S.E.M. ^*^p < 0.5, ^**^p < 0.01, ^***^p < 0.001 as calculated by two-tailed unpaired *t* test between the samples indicated.

During metastasis cancer cells are exposed to a poorly vascularized environment and serum starvation in cell culture can mimic such an environment *in vitro.* In both conditions, cancer cells undergo apoptosis unless they acquire resistance mechanisms such as activation of the PI3K/AKT cell survival pathway. In addition to environmental stress conditions, overexpression of constitutively active AKT also favors cell survival against Tamoxifen, ICI 182,780 and chemotherapy in breast cancer [57–59]. We have previously shown that knockdown of ZRF1 leads to cell death resistance in both, serum starved and drug treated, conditions (Figure 8A and 8C, Supplementary Figure 11A and 11B). Hence, we asked if compromised cell survival pathways are the reason for the decreased cell death observed in shZRF1 cells. To answer this question, we performed a time course experiment for TAM and ICI treatments following serum starvation for 48 hours. Western blot analysis of P-AKT (phosphorylated AKT) and AKT protein levels revealed that the PI3K/AKT pathway is activated in both serum starved and drug treated conditions (Figure 9A and 9B, Supplementary Figure 14C and 14D).

Cell survival requires the inhibition of apoptosis as well as the activation of signaling pathways such as the AKT pathway. BCL-2 and IAP (inhibitor of apoptosis) family members control the balance between the pro-apoptotic and anti-apoptotic genes which ultimately determines the cell fate [60–62]. Judged by the significant increase of the P-AKT levels in shZRF1 cells after ICI treatment (Figure 9B), we analyzed the expression of pro-apoptotic and anti-apoptotic genes belonging to the BCL-2 and IAP families in serum starved and ICI induced conditions (Figure 9C). While ZRF1 depletion increased the expression of all the anti-apoptotic genes tested significantly, the expression of pro-apoptotic genes remained unchanged in both conditions. Taken together, ZRF1 expression is probably necessary for the orchestration of cell death and cell survival related pathways in breast cancer cells. Furthermore, the balance between anti-apoptotic and pro-apoptotic genes seems impaired in the absence of ZRF1.

**Figure 10:**
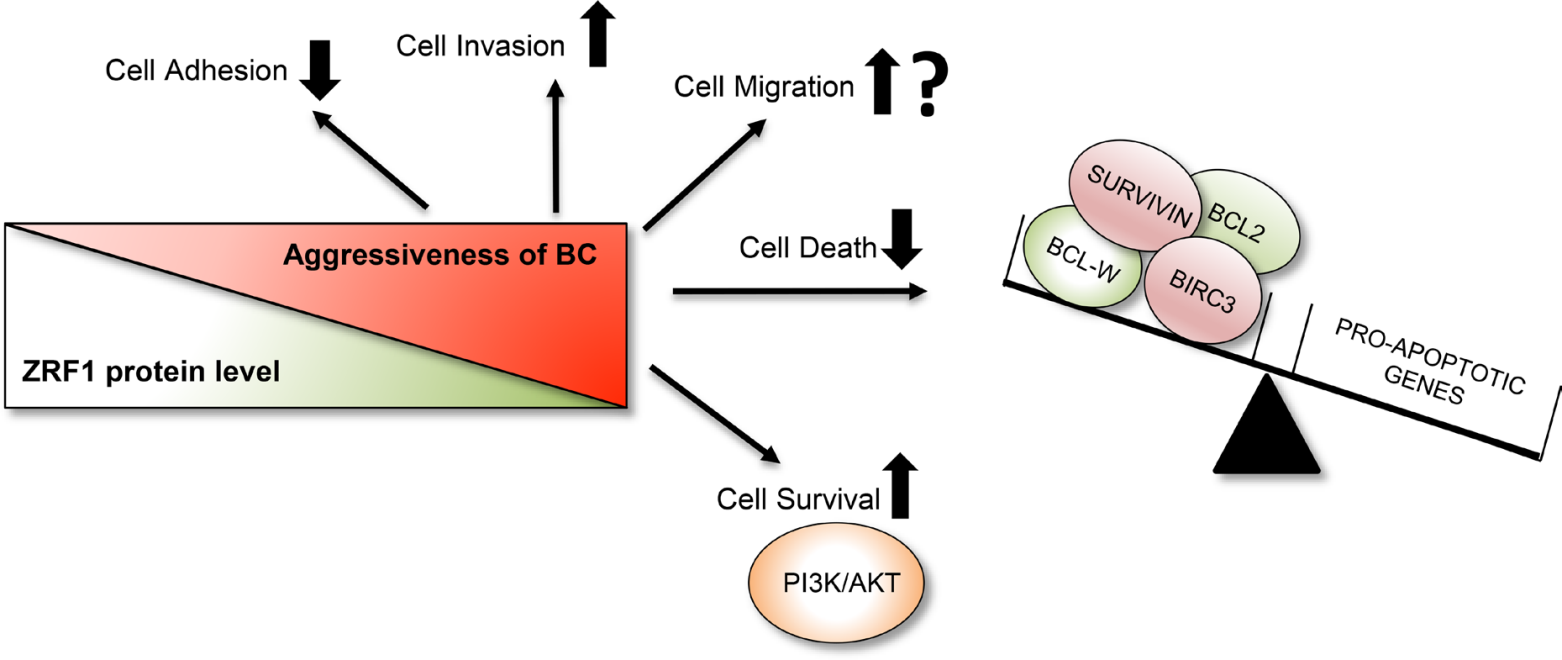
Summary of ZRF1’s role in breast invasive ductal carcinoma ZRF1 plays an important role in the progression of breast invasive ductal carcinoma into a more aggressive form. A decrease in ZRF1 protein levels results in decreased cell adhesion and significantly increased cell invasion capacities *in vitro*. ZRF1 has both stimulating and inhibiting effects on single cell migration depending on the experimental approach. However, in conditions where cells are migrating as clusters, such as in wound healing, ZRF1 increases cell motility. Furthermore, ZRF1 depletion contributes to drug resistance by decreasing cell death levels by disrupting the balance between anti-apoptotic and pro-apoptotic genes and by promoting cell survival through the PI3K/AKT pathway.

Based on our results, we propose that depletion of ZRF1 is one of the key players in the progression of breast carcinoma into a more metastatic and aggressive form of the disease. Our data illustrate that ZRF1 functions by controlling important biological processes such as cell adhesion, migration and invasion and the resistance of breast cancer cells towards current therapy options may be critically influenced by ZRF1 protein levels (Figure 10).

## DISCUSSION

The mortality rate of breast cancer reduced over the past 15 years due to advances in early detection and therapeutic regimens, yet the metastatic spread of advanced breast cancer remains the leading cause of death in women over the age 50 [63]. Hence, it is inevitable to understand the cellular and molecular mechanisms by which breast carcinoma develops and progresses into a metastatic stage in order to identify novel therapeutic approaches. In this study, we provide evidence that ZRF1 potentially acts as a tumor suppressor protein during the progression of breast invasive ductal carcinoma into a more advanced stage.

Depletion of ZRF1 has a strong effect on the acquisition of metastatic features in breast cancer in both 2D and 3D cultures. Our data suggests that a disrupted actin cytoskeleton together with improper cell polarity and decreased cell adhesion may facilitate the tumor cell dissemination from spheroids derived from shZRF1 cells [43, 64]. These defects in tissue integrity not only led to formation of loose spheroids, which were not capable of establishing tight connections with newly synthesized cells, but also stimulated already existing cancer cells to gain migratory and invasive behaviors. Knockdown of ZRF1 increased the invasion capacity of 3D spheroids significantly, which is regarded one of the hallmarks of metastasis. Interestingly, the same ZRF1 knockdown spheroids failed to migrate when compared to spheroids derived from control cells. One possible explanation for this observation is that depletion of ZRF1 enhances the proteolytic degradation of the basement membrane, which is a prerequisite for the migration into the surrounding tissue. Hence, shZRF1 cells may invade into a much larger area without increasing their migration capacity itself. In addition to cell migration and invasion, the cell proliferation capacity of spheroids derived from shZRF1 cells increased. This observation is in agreement with a previous report describing ZRF1 as a tumor suppressor that regulates the *INK4/ARF* locus epigenetically and protects genomic integrity during the cell cycle [43, 64]. However, we did not observe a similarly strong effect on cell growth in breast cancer cell lines when depleting ZRF1 compared to the dramatic growth phenotypes exhibited after ZRF1 knockdown in other cancer types [43, 65, 66]. Unlike for cell invasion, we noticed differences in cell proliferation and migration properties of shZRF1 cells when cultured in monolayer and in suspension. Different culture techniques are known to have a certain impact on cellular behavior [67–70], yet 3D culture systems provide a more realistic environment for the study of *in vivo* events [50]. Thus it is reasonable to assume that ZRF1 knockdown provokes a metastatic tumor phenotype by interfering with almost all the steps in the metastatic cascade. Still, further *in vivo* experiments are required to better understand the role of ZRF1 in breast cancer.

Metastasis is a complex process driven by different cellular networks in which EMT plays an initiative role. Loss of E-cadherin and expression of both N-cadherin and Fibronectin, which are considered hallmarks of EMT, lead to the gain of mesenchymal traits and metastasis of cancer cells [71]. Although the disrupted tissue integrity, the increased migration and invasion observed in shZRF1 cells point at an EMT phenotype, we did not notice the classical cadherin switch in these cells. Still, spheroids derived from shZRF1 MCF7 cells displayed an aggressive tumor phenotype with increased expression of almost all the EMT related TFs tested. These observations may suggest two different scenarios. In the first scenario, knockdown of ZRF1 in ER (+) cells would promote a mixed epithelial-mesenchymal phenotype [30], most probably due to the high heterogeneity of breast cancer cells [72]. Such a partial EMT might explain why shZRF1 cells are capable of migrating and invading effectively while still expressing epithelial markers such as E-cadherin as also previously reported [73–76]. Furthermore, shZRF1 cells can provoke an aggressive cancer phenotype as a mixed population of epithelial-mesenchymal cells facilitate a higher cell plasticity, as observed in clinically aggressive tumors [77, 78]. In the second scenario, knockdown of ZRF1 in ER (–) cells does not influence EMT simply because most of the relevant genes and signaling pathways are already misregulated. Likewise, ZRF1 could interfere with other signaling pathways or control the expression of genes involved in the metastatic cascade. For instance, ZRF1 plays a critical role in regulating WNT target genes during neural progenitor cell (NPC) differentiation. In ZRF1 depleted NPCs, canonical WNT signaling is impaired and the cellular levels of active β-catenin are decreased. A loss of gene expression in negative regulatory pathways may deregulate the WNT pathway in MDA-MB-231 cells. An aberrant activation of WNT/β-catenin signaling is associated with high grade TNBC with increased metastatic capacity and poor prognosis [79–82]. Collectively, ZRF1 might be controlling the expression of negative regulators of the WNT pathway in a similar manner as observed in NPCs.

Transcriptome analysis of spheroids derived from control and shZRF1 MCF7 cells reveal a central role for ZRF1 in breast cancer development. Particularly, mesoderm formation and inflammatory response related genes represent the two largest groups of genes which are affected by ZRF1 knockdown. With respect to the regulatory role of ZRF1 in mesoderm derived tissues in embryonic stem cells, it is comprehensive that genes important for mesenchymal cell differentiation and mammary gland development are mostly affected by ZRF1 knockdown. In line with previous reports, ZRF1 depleted cells also displayed aberrant differentiation towards adipose tissue when treated with retinoic acid [31, 43]. Our genomic data illustrate that knockdown of ZRF1 leads to an upregulation of inflammatory responses which are crucial for all stages of tumor development involving cancer initiation, promotion, malignant phenotype acquisition and metastasis. Chronic inflammation may influence the whole tumor organ by regulating the growth, migration and differentiation of all cell types in the tumor microenvironment, including neoplastic cells, fibroblasts and endothelial cells [83–85]. Hence, understanding how inflammatory responses and the tumor microenvironment are effected by ZRF1 depletion may be an intriguing focus of further research. In this regard, lipocalin-2 (LCN2) could be an interesting target to study. LCN2, whose expression is most upregulated (37.2 fold more compared to control) in shZRF1 MCF7 cells, was shown to promote breast cancer progression *in vitro* and *in vivo*, and it contributes particularly to early events of metastasis. Whereas LCN2-deficient mice significantly reduced tumor cell dissemination into the lung, neutralizing LCN2 antibody resulted in significant blockage of lung metastasis [86–88].

In addition to regulation of differentiation pathways, ZRF1 contributes to proper embryonic development by controlling cell migration such as controlling the correct radial migration during cerebral cortex development in mice [35]. Knockdown of ZRF1 in MCF7 and T47D cells dramatically augments cell migration in both wound healing and transwell migration assays. Although knockdown of ZRF1 in MDA-MB-231 cells did not recapitulate the increased wound healing capacity in MCF7 cells, overexpression of ZRF1 in the same cell line results in a significant decrease in wound healing. This observation might be rooted in the fairly low expression level of ZRF1 in MDA-MB-231 cells compared to MCF7 cells which makes the effect of ZRF1 knockdown in this cell line not as dramatic as in MCF7 cells. Taken together and based on our genomics data, we propose a key role for ZRF1 in controlling cell motility.

Unexpectedly, cell death related genes represent the largest group of genes affected upon ZRF1 knockdown in MCF7 cells. Depletion of ZRF1 strongly decreases apoptosis and promotes cell survival in breast cancer cells. ZRF1 knockdown induces the expression of anti-apoptotic genes (BCL-2, BCL-W, SURVIVIN and BIRC3) but does not affect the expression of pro-apoptotic genes after ICI 182,780 treatment. It is generally accepted that the balance between proliferation and apoptosis influences the response of tumors to treatments such as hormonal therapy, radiation and chemotherapy. Dysregulation of apoptosis is usually the reason why the treatment fails in the clinic. Hence, overexpression of BCL-2 upon ICI 182,780 treatment might be an important cause of the intrinsic resistance observed in shZRF1 cells. Furthermore, Tamoxifen treated ER (+) breast cancers often increase BCL-2 and BCL-XL levels which leads to a decreased acute tumor response and ultimately to a long-term resistance to Tamoxifen [22]. ZRF1 also controls the expression of IAPs in addition to anti-apoptotic BCL-2 family members. SURVIVIN is upregulated almost 20 fold more in shZRF1 cells upon ICI 182,780 treatment. The expression level of SURVIVIN is correlated with a more aggressive disease and poor clinical outcome [89, 90]. SURVIVIN plays also important roles in multidrug resistance in MCF7 cells [91] and may induce resistance in triple negative breast cancer cells by preventing apoptosis [92]. BIRC3 is another IAP family gene which is upregulated in shZRF1 cells after drug treatment. Studies have demonstrated that BIRC3 promotes resistance of cancer cells towards apoptosis [93, 94]. Furthermore, ZRF1 knockdown cells exhibit increased activation of the PI3K/AKT pathway which might be another reason for the observed endocrine resistance. The PI3K/AKT/mTOR pathway plays an important role in proliferation, differentiation and survival of cells and has been shown to be associated with resistance to endocrine therapy, human epidermal growth factor receptor 2 (HER2)-directed therapy and cytotoxic therapy in breast cancer [95]. Currently there are many ongoing studies on inhibitor drugs targeting the PI3K/AKT/mTOR pathway. Preclinical studies have demonstrated that the combination of mTOR inhibitors with endocrine therapy can help overcoming endocrine therapy resistance [96]. Further investigation addressing the interplay between ZRF1 and mTOR inhibitors might provide valuable information regarding the endocrine resistance in breast cancer cells. We also observed resistance to cell death in shZRF1 cells in serum starved conditions. It has been recently shown that nutrient sensing pathways impact on cell senescence through the phosphorylation of ZRF1 and the activation of mTORC1-S6Ks (mammalian target of rapamycin complex 1/ S6 kinases). Knockdown of ZRF1 or the expression of a ZRF1 phosphorylation mutant is sufficient to block the S6 kinase-dependent senescence program [64]. Hence, investigating the relationship between ZRF1, the mTOR pathway and the senescence program will help to better understand cell death regulation in breast cancer cells.

Taken together our data highlight an essential role for ZRF1 during breast cancer progression. Apart from the role of an transcriptional activator in stem cell differentiation, ZRF1 has the potential to function both as tumor suppressor and an oncogene in cancer. In this report, we have shown that a depletion of ZRF1 protein levels initiates a series of biological events which cause breast cancer cells to acquire metastatic features and to eventually become more aggressive tumors with endocrine resistance. Overall, ZRF1 holds the potential to be further explored for new treatment strategies in breast cancer, particularly for controlling the early events in metastasis.

## MATERIALS AND METHODS

### TCGA analysis

For genomic alteration and mRNA expression analysis, the publicly available cBioPortal software was used: http://www.cbioportal.org/ [46]. The TCGA breast cancer dataset from June 30th, 2016 was chosen for the analysis. In the software, putative copy number calls on 1080 cases were determined using GISTIC 2.0. Values: –2 = homozygous deletion; –1 = hemizygous deletion; 0 = neutral/no change; 1 = gain; 2 = high level amplification. Mutations were determined by whole exome sequencing. mRNA expression data were determined as mRNA z-scores (RNA Seq V2 RSEM). The link for the aforementioned analysis is shown in the following: http://www.cbioportal.org/index.do?session_id=5abcb604498eb8b3d5656c7a.

### Preparation and usage of chemical stocks

4-OH Tamoxifen (H7904) was purchased from Sigma. ICI 182,780 (Cat No. 1047) was purchased from TOCRIS. All chemicals were prepared in 100% ethanol as 1000× stocks in the following concentrations: 4-OH Tamoxifen 10 mM, ICI 182,780 either 1 mM or 100 μM. For flow cytometry and western blot experiments, 4-OH Tamoxifen was used at a final concentration of 10 µM for 48 hours. For flow cytometry experiments, ICI 182,780 was used at a 100 nM concentration during 7 days. Every 2 days, medium was replenished. For western blot and real time qPCR experiments, ICI 182,780 was used at a 1 µM concentration for 48 hours. The same final concentrations of vehicle (100% ethanol) were used in all control experiments. Cisplatin was purchased from European Pharmacopeia Reference Standard and was prepared freshly in saline solution for every experiment. For flow cytometry and western blot experiments, cisplatin was used at a 100 µM concentration for 24 hours. The same volume of vehicle (saline) was used in all the control experiments.

### Cell culture

MCF7, MDA-MB-231 and MDA-MB-453 cells were a kind gift from Dr. George Reid. T47D cells were obtained from the IMB Flow Cytometry Core Facility. HEK293T cells were obtained from the American Type Culture Collection (Rockville, MD, USA). MCF7, MDA-MB-231, MDA-MB-453 and HEK293T cells were maintained in DMEM (Gibco) supplemented with 10% fetal bovine serum (Gibco), L-Glutamine (Gibco) and Penicillin/Streptomycin (Gibco). T47D cells were maintained in RPMI (Gibco) supplemented with 10% fetal bovine serum (Gibco), L-Glutamine (Gibco) and Penicillin/Streptomycin (Gibco). Prior to Tamoxifen or ICI 182,780 treatments, MCF7 cells were washed 2 times with 1xPBS and cultured in phenol red free DMEM supplemented with 5% charcoal-stripped FBS (Sigma-F6765), L-Glutamine (Gibco) and Penicillin/Streptomycin (Gibco) for 48 hours. During the starvation, the medium was changed every day.

### Transfection and lentiviral infection

For the production of lentiviruses containing shRNA, HEK293T cells were grown to 80% confluency and roughly 2 × 10^6^ cells were transfected by the PEI method using 2 μg pMDLg/pRRe, 2 μg pRSV-Rev, 2 μg pMD2.G and 4 μg shRNA#55 (TRCN0000254055) or shRNA#58 (TRCN0000254058) plasmids. 48 hours post-transfection, the medium containing the viral particles was collected and added to 2 × 10^5^ MCF7/ T47D/ MDA-MB-231/ MDA-MB-453 cells in a 6-well plate for transduction. After repeating the same procedure, cells were left to recover for 48 hrs. Knockdown cells were kept in constant selection with 2 μg/ml puromycin (Sigma). For overexpression experiments, the pEV833-GFP-ZRF1 plasmid [43] was used, which was transfected into MDA-MB-231 cells as described above. After isolation of GFP positive single cell derived clones, the most prominent clone was used for further experiments.

### Cell growth analysis

For cell growth experiments, 1 × 10^4^ cells were seeded into 24-well plates in 9 replicates: a pool of 3 wells was considered one technical replicate. Cells were counted on every second day with a hemocytometer. The medium was changed every second day.

### Cell adhesion

Collagen coated 24-well plates (ZenBio) were blocked with 1%BSA dissolved in DMEM. Either 2 × 10^5^ MCF7 cells or 5 × 10^5^ T47D cells suspended in 1ml DMEM (no supplements) were added to each well in 4 replicates. Cells were allowed to attach to the plate surface for 1 hour (MCF7) or 3 hours (T47D) at 37° C, and then fixed with 4% PFA (paraformaldehyde) for 10 min at room temperature. After staining with crystal violet (5 mg/ml) for 10 min, microscopy images were taken with a Leica DM-IL microscope. Crystal violet was dissolved in 33% acetic acid and its absorbance was measured at 550 nm in a TECAN plate reader. Cell adhesion capacity was calculated as fold change after background normalization of absorbance values.

### Wound healing

Cells were grown to 100% confluence in 6-well plates and the cell monolayer was scraped in a straight line with a p200 pipet tip to create a wound. For MCF7, T47D and MDA-MB-453 cells images were taken every 24 hours during 72 hours with a Leica DM-IL microscope. For MDA-MB-231 cells images were taken at 0, 4, 8, 12 and 24 hours with a Leica DM-IL microscope. The wound width at each time point was calculated with the ImageJ software and relative wound width was calculated after normalization of each value in relation to the 0 time point of each cell line using the same software.

### Transwell cell migration

For transwell experiments in MCF7 cells, 96-well transwell migration plates (Corning-Ref: CLS 3384) were used. While the reservoir plate was filled with attractant medium (DMEM supplemented with 20% FBS), 2 × 10^4^ cells in DMEM without FBS were seeded into the inner plate. Cells were left for migration for 24h and then fixed in 3.7% formaldehyde in 1xPBS for 2 min at room temperature. For transwell experiments in T47D cells, 24-well transwell inserts (Corning-Ref: 3464) were used. 1 × 10^5^ cells in DMEM without FBS were seeded into each insert and the reservoir plate was filled with the same attractant medium. Cells were left for migration for 48h and then fixed in the same way. After crystal violet (1%) staining for 15 min, the transwell plate/ insert was washed and the remaining stains in the upper part of the transwell/ insert were wiped off with Kimtech paper. Images of migrated cells on the lower part of the transwell were acquired with a Leica DM-IL microscope. For MCF7 cells, total numbers of migrated cells from 12 wells of a 96-well transwell plate, for T47D cells, total numbers of migrated cells from 3 inserts of 24-well transwell inserts were calculated and represented as fold change of migration.

### Transwell cell invasion

The invasion assay was identical to the above migration assay except that transwell plates or inserts were coated with Matrigel (Corning-Ref: 356234) diluted to 200 µg/mL. For 96-well plates 20 µL, for 24-well inserts 100 µL of diluted Matrigel was used for coating. The experiment was stopped after 72 h using the same method as described above and represented as fold change of cell invasion.

### 3D spheroid culture

Regular tissue culture 96-well plates were covered with 1% agarose dissolved in 1xPBS. MCF7 or T47D cells were prepared at a 5 × 10^6^ concentration in regular DMEM and 100 μL of cell suspension was added to each well of 96-well plates. Cells were left in suspension to generate spheroid like structures for 7 days. Every 2 days, medium was replenished. Images were acquired with a Leica DM-IL microscope. The surface area of each spheroid was calculated using the ImageJ software.

### Tumor-spheroid based migration assay

Tumor-spheroid based migration assay was performed as published with minor changes [51]. Briefly, 1 × 10^3^ MCF7 cells per well were dispensed into ultra-low attachment 96-well plates (Corning-Ref:7007) and incubated for 5 days. Flat-bottomed, 96-well plates were coated with 0.1% (v/v) gelatin (Sigma) in sterile water for 1 h at 37° C. A total of 200 μl/well of culture medium supplemented with 2% (v/v) FBS was then added. 5 days old spheroids were transferred into the prepared ‘migration’ plate and were allowed to adhere. Images were acquired with a Leica DM-IL microscope. The total area of each spheroid was measured at days 5 and 7 using the ImageJ software.

### Tumor-spheroid based Matrigel invasion assay

The tumor-spheroid based Matrigel invasion assay was performed as published with minor changes [51]. 3D spheroids derived from MCF7 cells were generated as mentioned above. Then 5 days old spheroids were embedded in 100 μl growth factor reduced Matrigel (Corning-Ref: 354230) in each well of a µ-Slide 8 Well (ibidi). After Matrigel had solidified, 100 μl of normal growth medium was added on top of the Matrigel and spheroids were allowed to invade for 72 hours. Invasion efficiency was calculated as percentage ratio of spheroids which showed Type I or Type II invasion to total number of spheroids. The invasion area of each spheroid was calculated using the ImageJ software.

### BrdU incorporation and cell cycle

For normal cell cycle analysis, MCF7 or T47D cells were treated with 10 µM of BrdU solution for 30 min. After denaturation, samples were incubated with DAPI for 30 min following either 1 hour of Anti-BrdU-488 AlexaFluor (Biolegend: Ref 364106) incubation for MCF7 cells or 1 hour of Anti-BrdU-647 AlexaFluor (Biolegend: Ref 364108) for T47D cells. 7 days old spheroids were treated with 0.25 mg/ml of BrdU solution for 60 min, dissociated into single cells and fixed with 80% ethanol overnight at −20° C. After denaturation with HCl and samples were incubated with Anti-BrdU-488 AlexaFluor (Biolegend) for 1 hour at RT. The percentage of BrdU-positive cells was analyzed by flow cytometry on a BD LSR Fortessa. For the cell cycle analysis in ZRF1 overexpressing MDA-MB-231 cells, Click-IT technology was used (Thermo Fischer-Ref C10634). Cells were treated with 10 µM of EdU solution for 30 min and experiments were conducted according to kit’s manual. For each experiment at least 10000 events were counted. Cell cycle distributions were calculated with the FlowJo program.

### RNA sequencing

For each replicate, RNA was isolated from 7 days old spheroids derived from control and shZRF1 MCF7 cells using Direct-zol RNA MiniPrep kit (Zymo Research). NGS library preparation was performed with Illumina’s TruSeq stranded RNA LT Sample Prep Kit following Illumina’s standard protocol (Part # 15031048 Rev. E). Libraries were prepared with a starting amount of 900 ng and amplified in 10 PCR cycles. Libraries were profiled in a High Sensitivity DNA on a 2100 Bioanalyzer (Agilent technologies) and quantified using the Qubit dsDNA HS Assay Kit, in a Qubit 2.0 Fluorometer (Life technologies). All 6 samples were pooled in equimolar ratio and sequenced on 1 NextSeq Highoutput Flowcell, SR for 1× 85 cycles plus 7 cycles for the index read. Libraries were sequenced on a Illumina NextSeq 500, with an average depth of 72M reads per sample. Reads were mapped to the Human Ensembl GRCh38 version 84 reference genome [97] using STAR [98] version 2.5.2b, allowing up to 2 mismatches, a minimum intron length of 21, discarding reads mapping to more than 10 loci, and eventually keeping only the primary alignment. Quality control of the sequenced reads was done with FastQC, dupRadar [99] and other in-house developed tools. We then counted reads on genes using featureCounts [100] from the Subread package version 1.5.1 and using the gene model provided by Ensembl for the same assembly version (GRCh38 version 84). We did the differential expression analysis with DESeq2 [101] version 1.14.1, with the default Wald test to calculate the significance. The GO enrichment analysis was done using the clusterProfiler package for R. Additional gene sets were obtained from the Molecular Signatures Database [102] v6.0 from the Broad Institute. All the information including the RNA-seq data is listed in the Supplementary Tables 2–4. The sequencing data was deposited to the GEO repository under GSE104675.

### Immunofluorescence staining

For Phallodin staining, MCF7 cells were grown on coverslips and fixed with 4% PFA for 10 min at RT. After permeabilization with 0.1% Triton in 1xPBS for 5 min on ice, cells were blocked in 1%BSA in 1xPBS (blocking solution) for 30 min at RT. Subsequently coverslips were incubated with Phalloidin-Atto 565 (Sigma) for 30 min at room temperature, mounted with VECTASHIELD Antifade Mounting Medium with DAPI (Vector) and sealed. Fluorescence images were acquired on a Leica SP5 microscope. For E-cadherin staining, 7 days old spheroids were fixed in 4% PFA for 30 minutes, treated with sucrose, embedded in OCT and frozen at –80° C. The next day frozen blocks were sectioned at 5 μm thickness. Coverslips were incubated with 1:50 dilution of E-cadherin (AF748-Bio Techne) antibody for overnight at 4° C and secondary antibody (Alexa Fluor 488) for 1.5 hours at RT.

### Cell differentiation

Cell differentiation experiments were performed as published [55] with minor changes. Briefly, MCF7 cells grown on cover slips were treated with 10 µM *all-trans*-Retinoic acid (ATRA) (Sigma) for 96 hours. The same volume of DMSO was added to the cell culture medium as vehicle control. The cell culture medium was replenished after 48 hours. After 96 hours, coverslips were fixed with 4% PFA for 20 min at RT. After permeabilization with 0.5% Triton in 1xPBS, lipid droplets were stained with 1/2000 dilution of a Nile Red stock (1 mg/ml in 1xPBS) for 5 min and mounted with Fluoromont G. Lipid droplets were counted using the ImageJ software.

### RNA extraction, cDNA synthesis and real time qPCR

RNA used for qPCR was extracted with Trizol. 1 μg of total RNA was used for cDNA, utilizing the First Strand cDNA Synthesis Kit (Fermentas). The primers used in the reverse transcription qPCR assays are listed in Supplementary Table 1.

### Western blot

Cell pellets were collected and resuspended in 2x Laemmli buffer according to the pellet size. Subsequently probes were sonicated for 15 cycles (30 sec on/ 30 sec off) at high setting using a Diagenode Bioruptor plus. After boiling for 20 min at 95° C, samples were centrifuged for 15 at RT maximum speed and separated on 10% SDS-PAGE for Western Blot analysis. ZRF1 (Homemade), H2B (#12364-Cell Signaling), α-Tubulin (AB15708-Sigma), cleaved PARP (ab32064-Abcam), H2B (#12364-Cell Signaling)AKT (#9272S-Cell Signaling) and P-AKT (#9271T-Cell Signaling) antibodies were used to detect the respective proteins. Relative band intensities were calculated by using the Image Lab program from Bio-Rad.

### Trypan blue counting and apoptosis

To assess the cell death number under normal conditions, MCF7 or T47D cells were stained with trypan blue and counted under a light microscope during 10 days. Results were represented as percentage (%) of dead cells. Apoptosis analysis was performed by double staining with either Annexin V-FITC (BioLegend-Ref: 640906) for MCF7 cells or Annexin V-AlexaFluor647 (Biolegend-Ref: 640912) for T47D and MDA-MB-231 cells together with Propidium Iodide (PI) (Sigma) according to the manufacturer’s protocol. After staining, cells were analyzed by flow cytometry on a BD LSR Fortessa. For each experiment at least 10000 events were counted. Apoptosis analysis was calculated with the FlowJo program.

### MTT assay

Prior to the MTT assay, MDA-MB-231 cells were seeded at a density of 1 × 10^5^ cells per well of 48 well plates in 8 replicates. On the next day increasing amounts of Cisplatin were added to cells and incubated for 24 h. MTT (Sigma) powder was dissolved in 1xPBS at a concentration of 5 mg/ml, added as 1/10 volume of the cell culture medium volume and incubated at 37° C for 4 hours in the dark. After incubation, medium was collected carefully and 0.04 N HCl in isopropanol was added to each well to dissolve the formazan crystals. Absorbance was measured at 570 nm in a TECAN plate reader. Cell viability was calculated according to the control samples after subtraction of background absorbance.

## SUPPLEMENTARY MATERIALS FIGURES AND TABLES










