# Supplementary material for: Role for the transcriptional activator ZRF1 in early metastatic events in breast cancer progression and endocrine resistance
Source: Oncotarget. 2018 Jun 19;9(47):28666–90. doi: 10.18632/oncotarget.25596 (PMC6033359; doi:10.18632/oncotarget.25596)
Supplement: Supplementary file 1 [file oncotarget-09-28666-s001.pdf]

Role for the transcriptional activator ZRF1 in early metastatic events in breast cancer progression and endocrine resistance

SUPPLEMENTARY MATERIALS

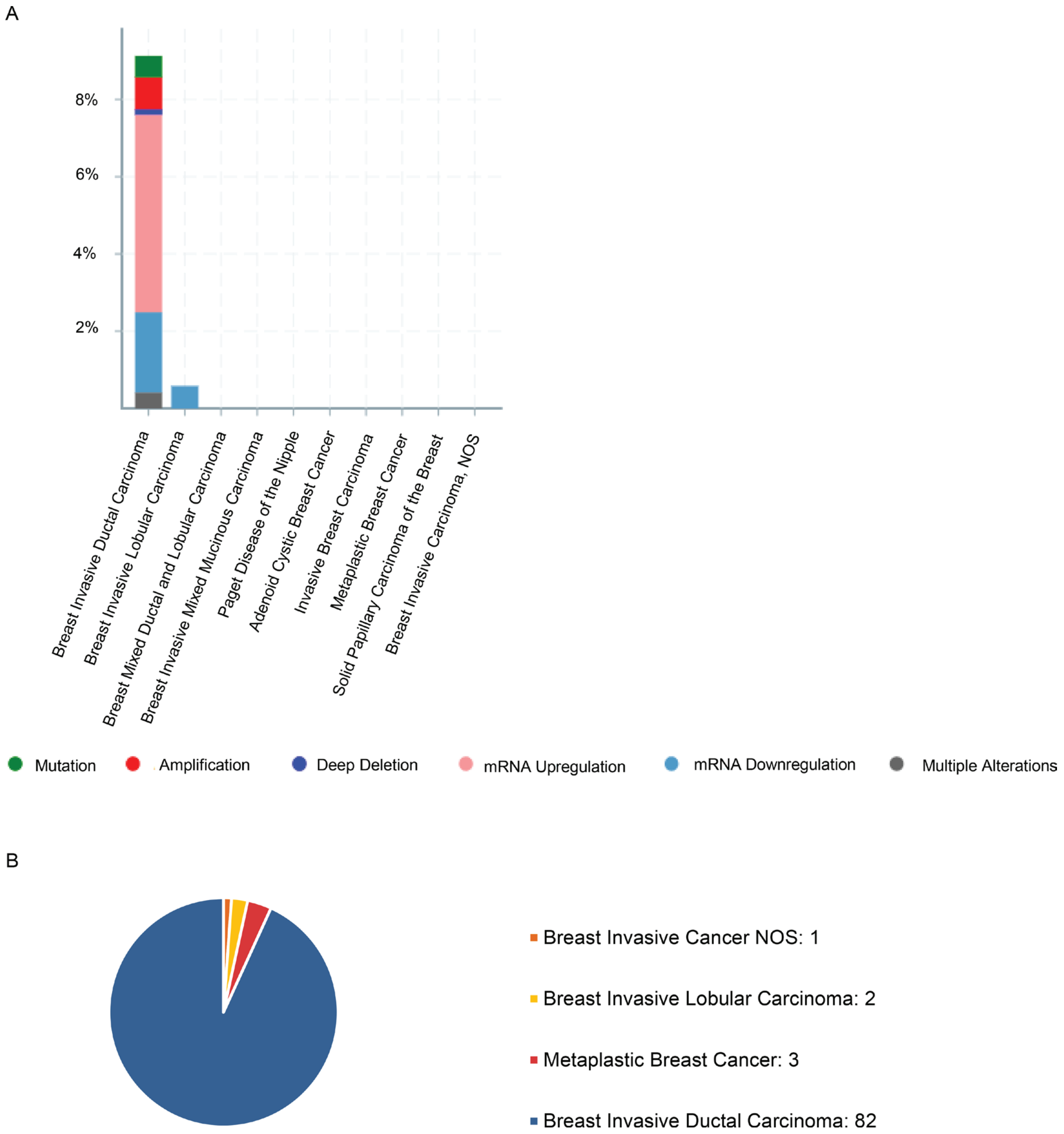

Supplementary Figure 1: ZRF1 is mostly associated with breast invasive ductal carcinoma. (**A**) Alteration frequency analysis in TCGA dataset (June 30th, 2016 dated) points at a specific role for ZRF1 in breast invasive ductal carcinoma. (**B**) mRNA expression analysis of the same data set summarizes the distribution of RNA level changes of ZRF1 in different type of breast cancers.

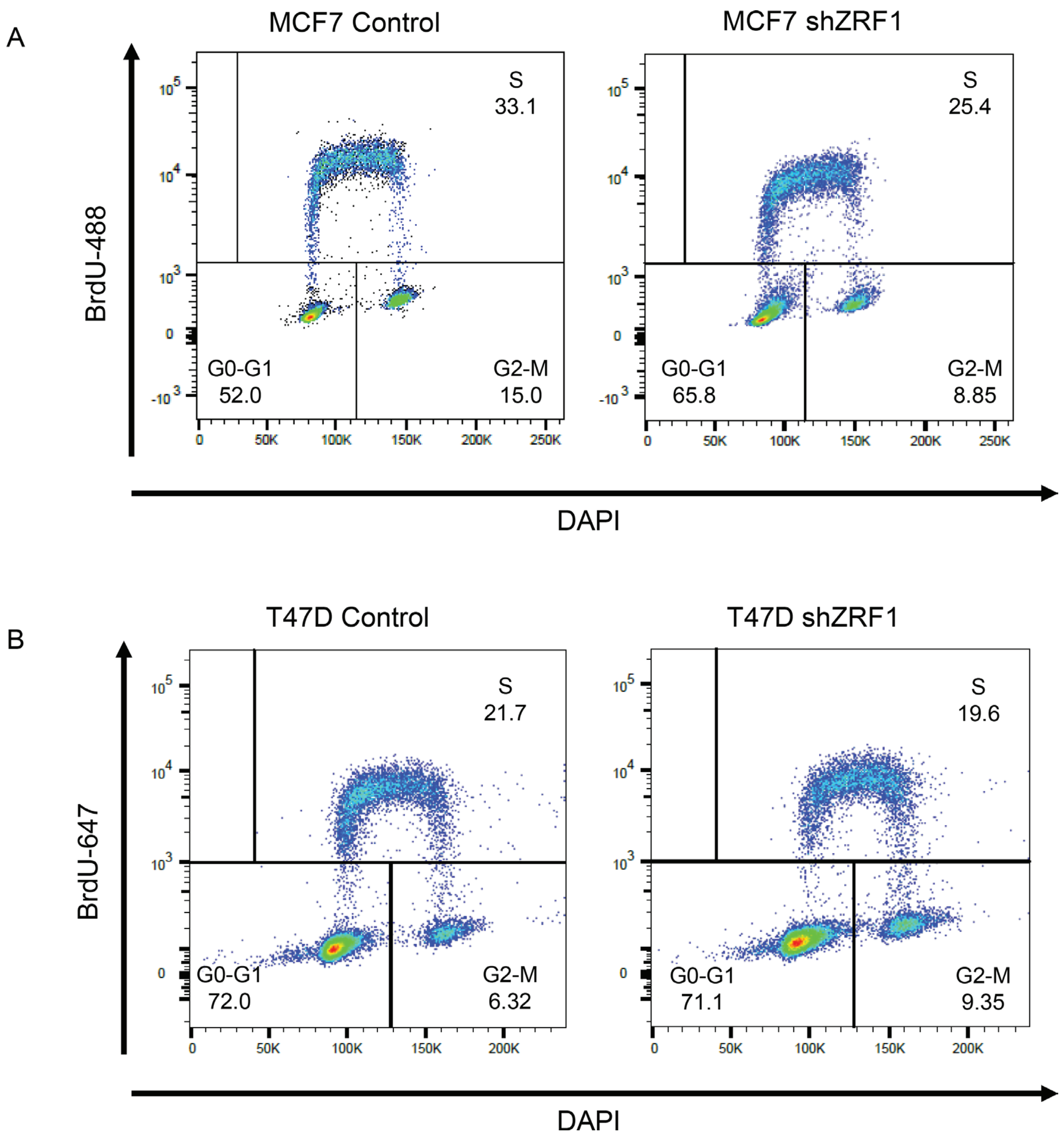

Supplementary Figure 2: ZRF1 knockdown leads to decreased cell numbers in S phase in both MCF7 and T47D cells. (**A**) Representative flow cytometry analysis of cell cycle distribution of control and shZRF1 MCF7 cells after BrdU-488 and DAPI double staining. (**B**) Representative flow cytometry analysis of cell cycle distribution of control and shZRF1 T47D cells after BrdU-647 and DAPI double staining.

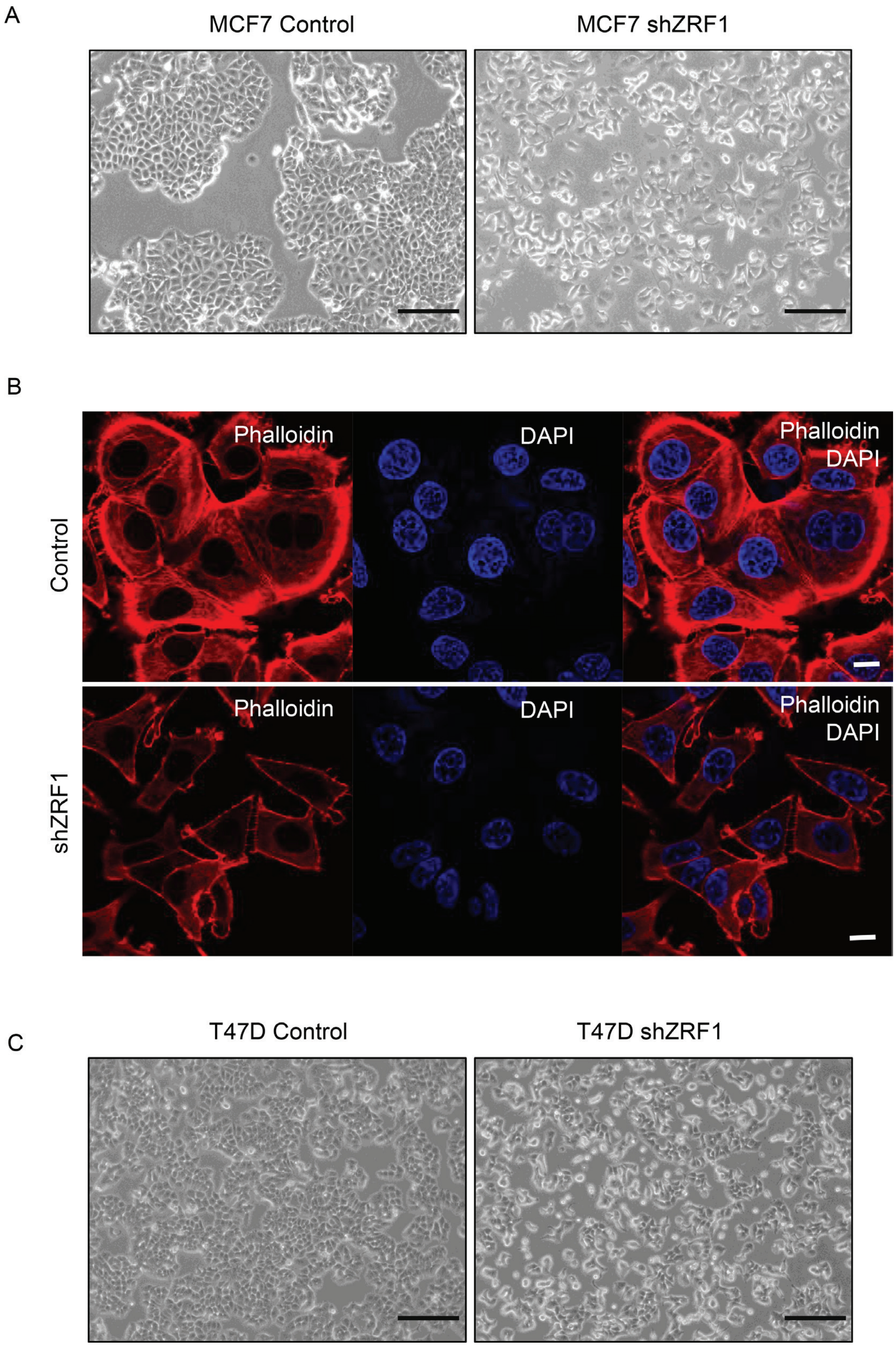

Supplementary Figure 3: ZRF1 depletion alters cellular morphology and decreases cell-cell interactions in both MCF7 and T47D cells. (**A**) Brightfield images of control and shZRF1 MCF7 cells were taken at 10× magnification. Scale bar, 200 µm. (**B**) Representative immunofluorescence images of Phalloidin staining in control and ZRF knockdown MCF7 cells. Scale bars, 5 µm. (**C**) Brightfield images of control and shZRF1 T47D cells were taken at 10× magnification. Scale bar, 200 µm.

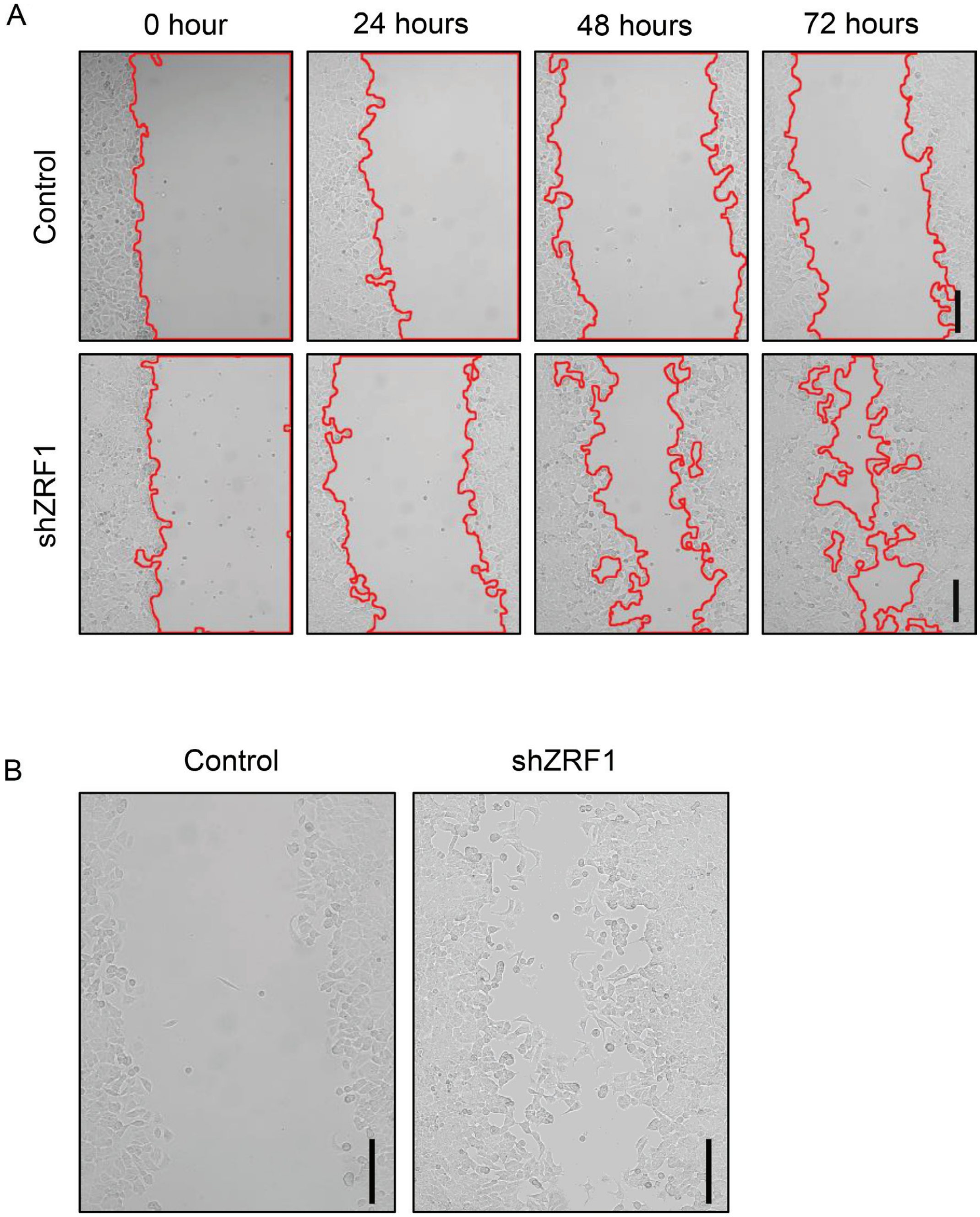

Supplementary Figure 4: ZRF1 depleted MCF7 cells preserve their spindle like morphology during wound healing assay. (**A**) Wound healing assays of control and shZRF1 cells during 72 hours. Images were taken at 10× magnification every 24 hours. Scale bar, 200 µm. (**B**) Brightfield images of control and shZRF1 derived MCF7 cells 72 hours after scar tissue generation indicate spindle like cell morphology in ZRF1 knockdown cells.

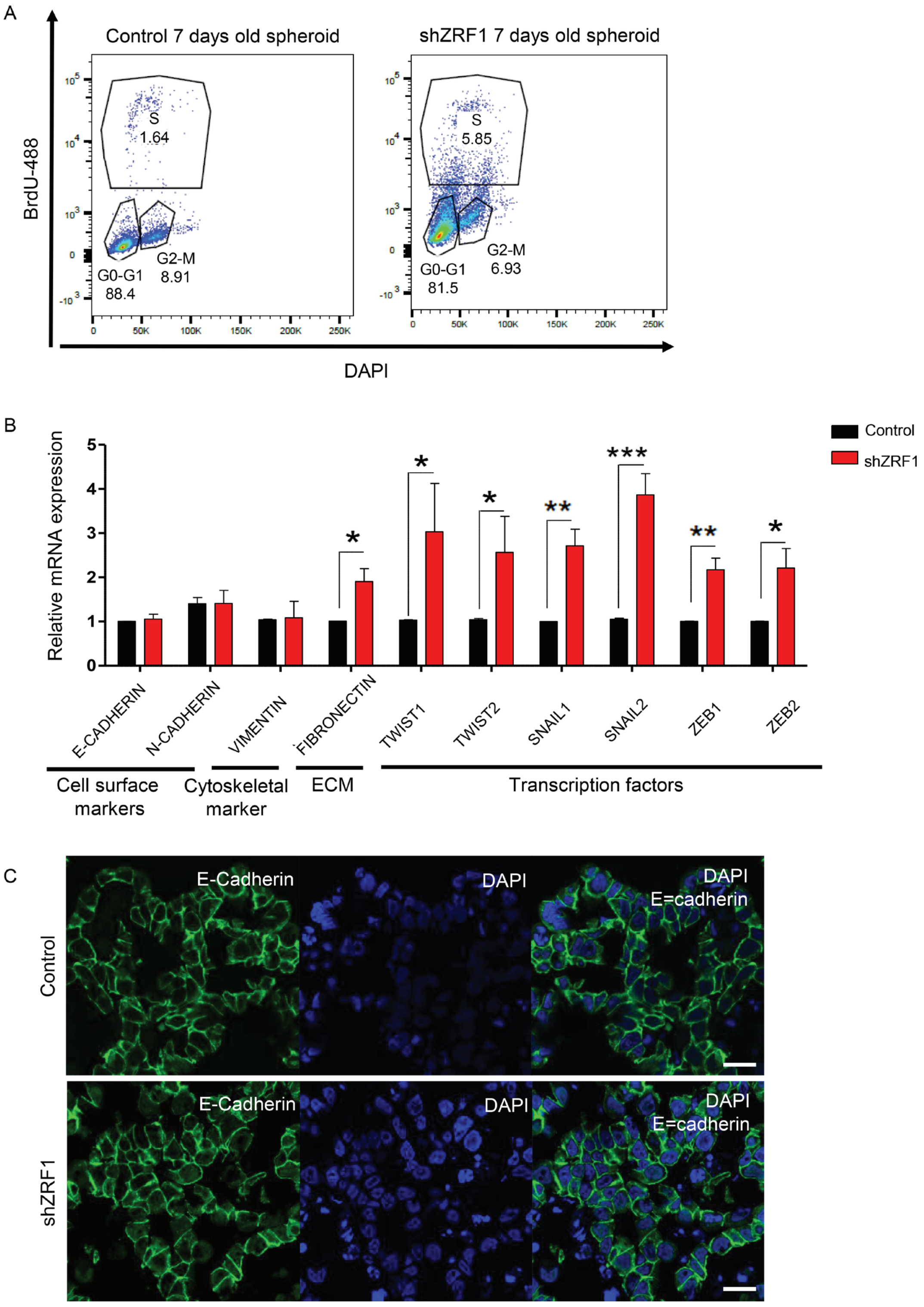

Supplementary Figure 5: ZRF1 knockdown cells are capable of generating spheroids prone to metastasis. (**A**) Representative flow cytometry analysis of cell cycle distribution of the single cells dissociated from 7 days old spheroids derived from control and shZRF1 cells after BrdU-488 and DAPI double staining. (**B**) Real-time qPCR of EMT related genes in the single cells dissociated from 7 days old spheroids derived from control and shZRF1 cells. Expression was normalized to the housekeeping gene GAPDH. Data represent the average of three experiments, ± S.E.M. $^{*}p < 0.5$, $^{**}p < 0.01$, $^{***}p < 0.001$ as calculated by two-tailed unpaired *t* test between the samples indicated. (**C**) Representative immunofluorescence images of E-cadherin staining of cryosections of 7 days old spheroids derived from control and shZRF1 cells. Scale bars, 20 µm.

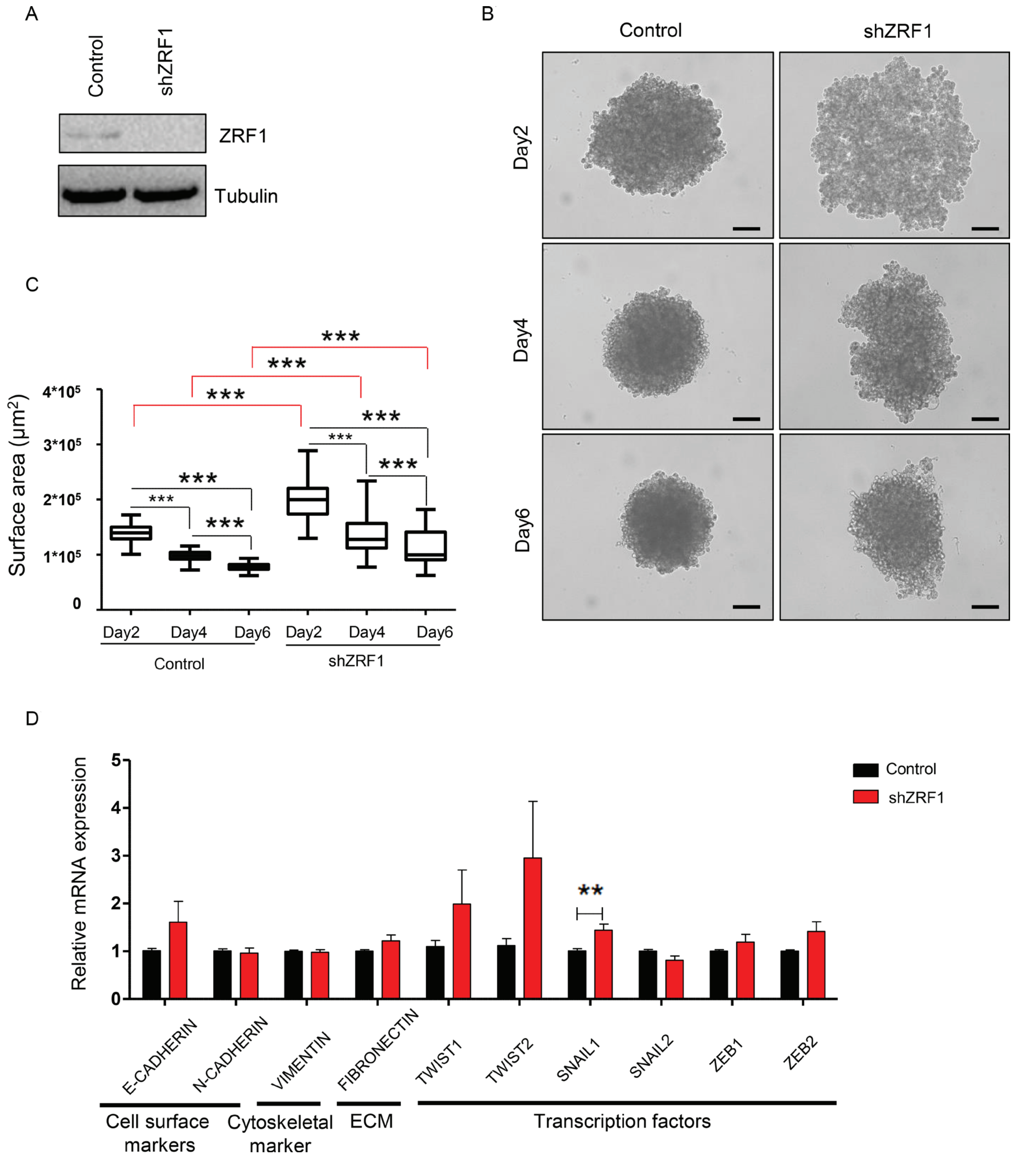

Supplementary Figure 6: ZRF1 depleted MDA-MB-231 cells exhibit a loose spheroid phenotype with decreased amount of cellular interaction. (**A**) Western blot for ZRF1 after viral induction of MDA-MB-231 cells. Alpha tubulin was used as a loading control. (**B**) Brightfield images of the spheroids derived from control and shZRF1 MDA-MB-231 cells were taken at 10× magnification at days 2, 4 and 6. Scale bars, 100 µm. (B) Quantification of the surface area of the spheroids derived from control and ZRF1 knockdown cells during 6 days. The surface area of the each spheroid was calculated using the ImageJ software. The whiskers of the plots represent the minimum and maximum values of the population of counted spheroids. $^{***}p < 0.001$ as calculated by two-tailed unpaired *t* test. (**C**) Real-time qPCR of EMT related genes in single cells dissociated from 7 days old spheroids derived from control and shZRF1 cells. Expression was normalized to the housekeeping gene GAPDH. Data represent the average of three experiments, ± S.E.M. $^{**}p < 0.01$, as calculated by two-tailed unpaired *t* test between the samples indicated.

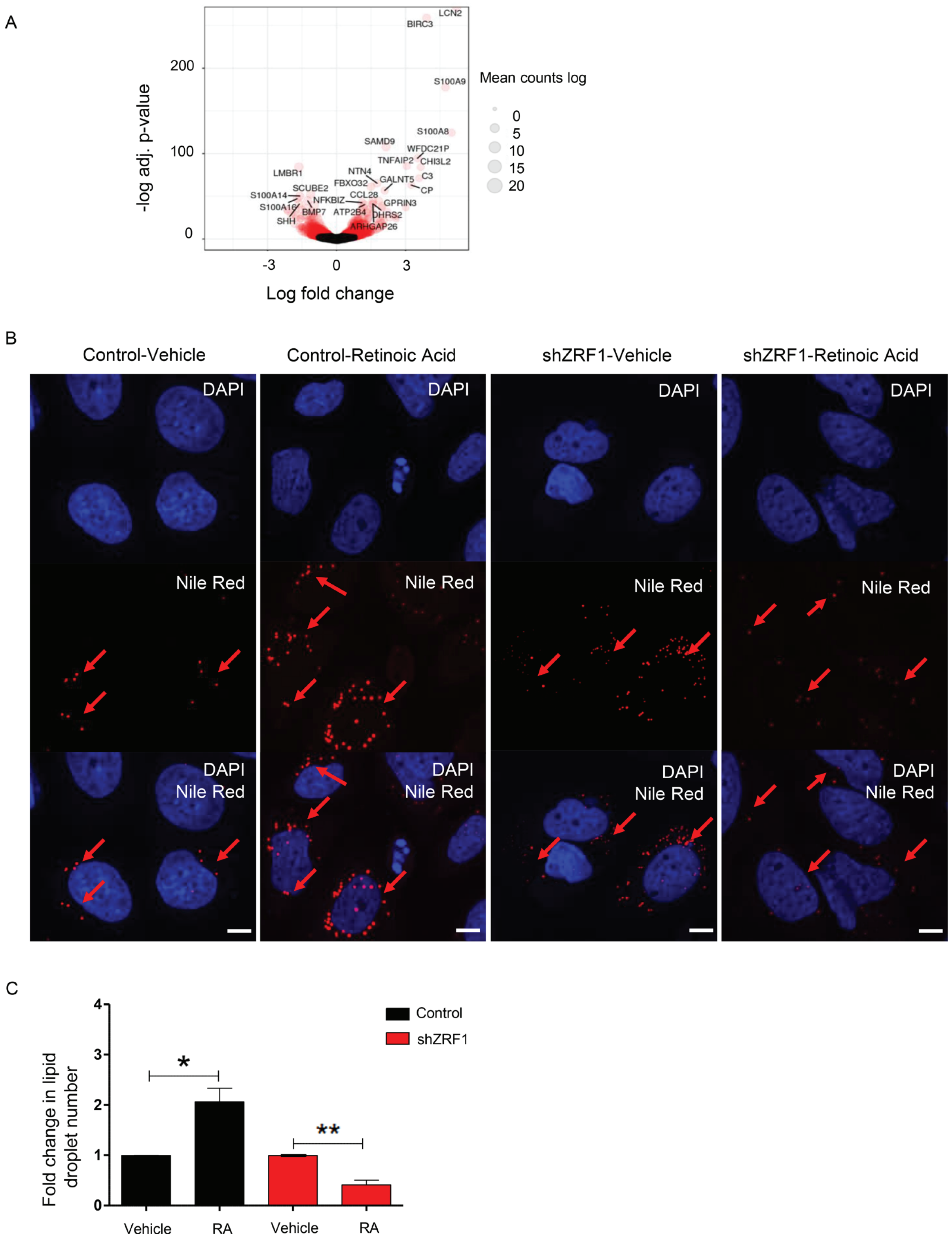

Supplementary Figure 7: The differentiation potential of ZRF1 knockdown MCF7 cells is impaired. (**A**) The volcano plot shows expression differences between the experimental conditions (x-axis, log2 fold change) and statistical significance (y-axis, $-\log_{10}$ adjusted p-values using the Benjamini & Hochberg method). Genes with FDR < 0.01 are highlighted in red, and the top 25 in terms of effect size are labeled. (**B**) Representative immunofluorescence images of Nile Red staining after 4 days of vehicle or retinoic acid treatment. Scale bars, 10 µm. (**C**) Quantification of the lipid droplets by each cell line was calculated as “Total Nile Red Positive Lipid Droplet Number/ Total Nucleus” and represented as fold change in lipid droplet number compared to their control conditions. Data represent the average of three experiments, ± S.E.M. $^{*}p < 0.5$, $^{**}p < 0.01$ as calculated by two-tailed unpaired t test between the samples indicated.

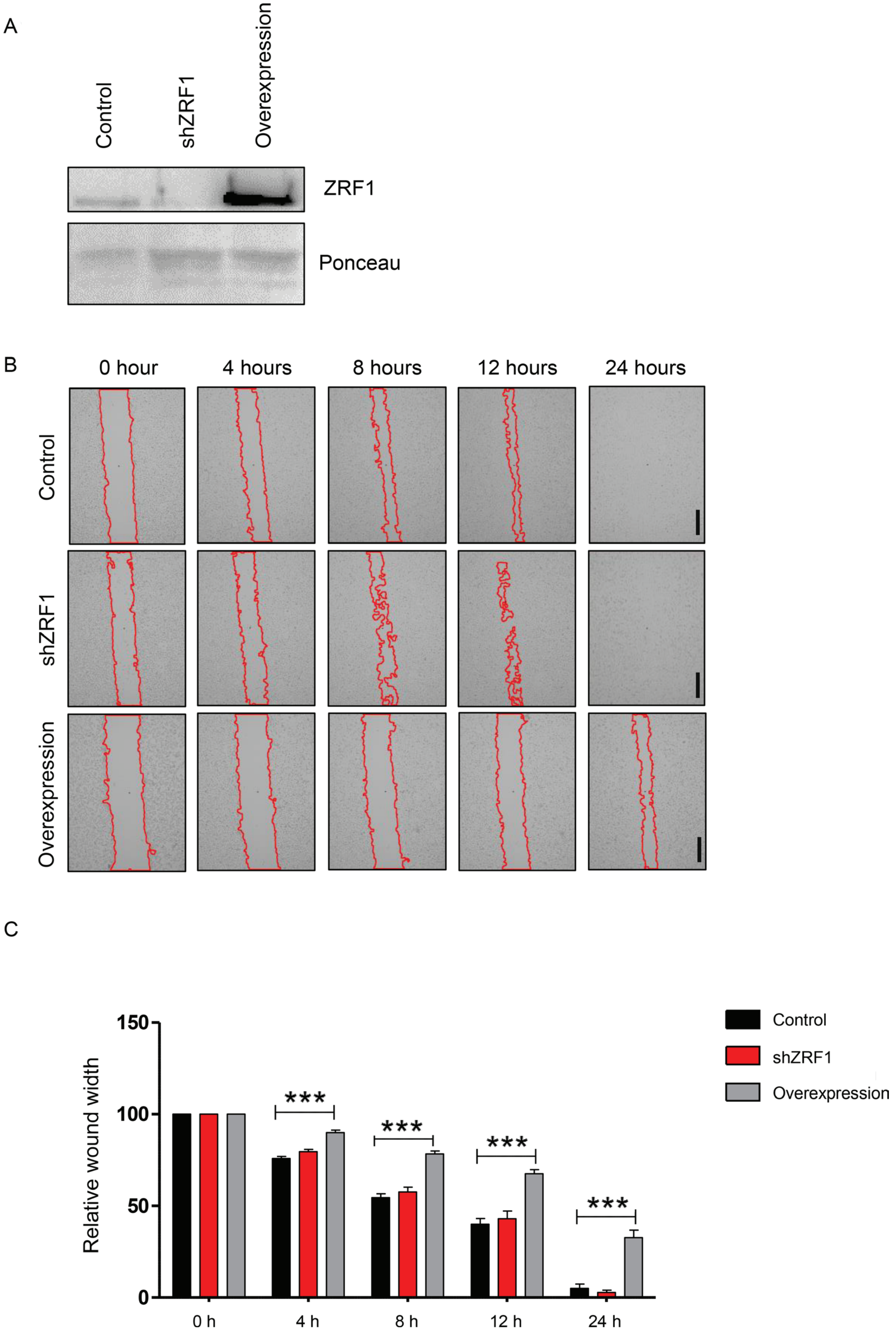

Supplementary Figure 8: Overexpression of ZRF1 diminishes the cell motility in MDA-MB-231 cells. (**A**) Western blot for ZRF1 after transfection with an ZRF1 overexpression plasmid. Ponceau was used as a loading control. (**B**) Wound healing assays of control, shZRF1 and ZRF1 overexpressing cells. Images were taken at 4, 8, 12 and 24 hours after the formation of a scar tissue. Scale bar, 500 μm. (**C**) Quantification of wound healing was calculated as relative wound width after normalization of each value in relation to the 0 time point of each cell line. Data represent the average of three experiments. ± S.E.M. $^{***}p < 0.001$ as calculated by two-tailed unpaired *t* test between the samples indicated.

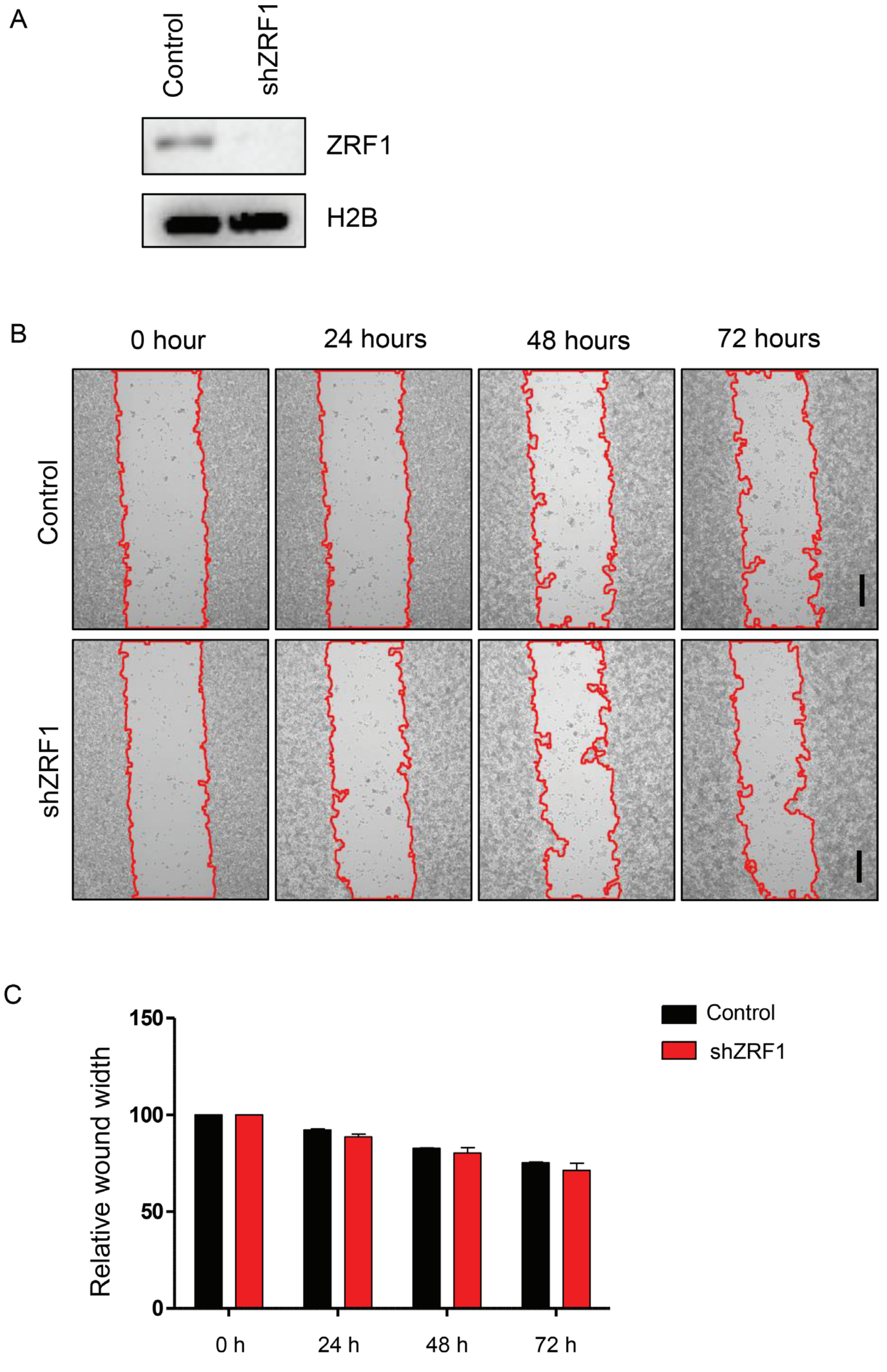

Supplementary Figure 9: Cell motility of ZRF1 depleted MDA-MB-453 cells. (**A**) Western blot for ZRF1 after viral induction of MDA-MB-453 cells. Histone H2B was used as a loading control. (**B**) Wound healing assays of control and shZRF1 cells. Images were taken at 24, 48 and 72 hours after the formation of a scar tissue. Scale bar, 200 µm. (**C**) Quantification of wound healing was calculated as relative wound width after normalization of each value in relation to the 0 time point of each cell line. Data represent the average of three experiments.

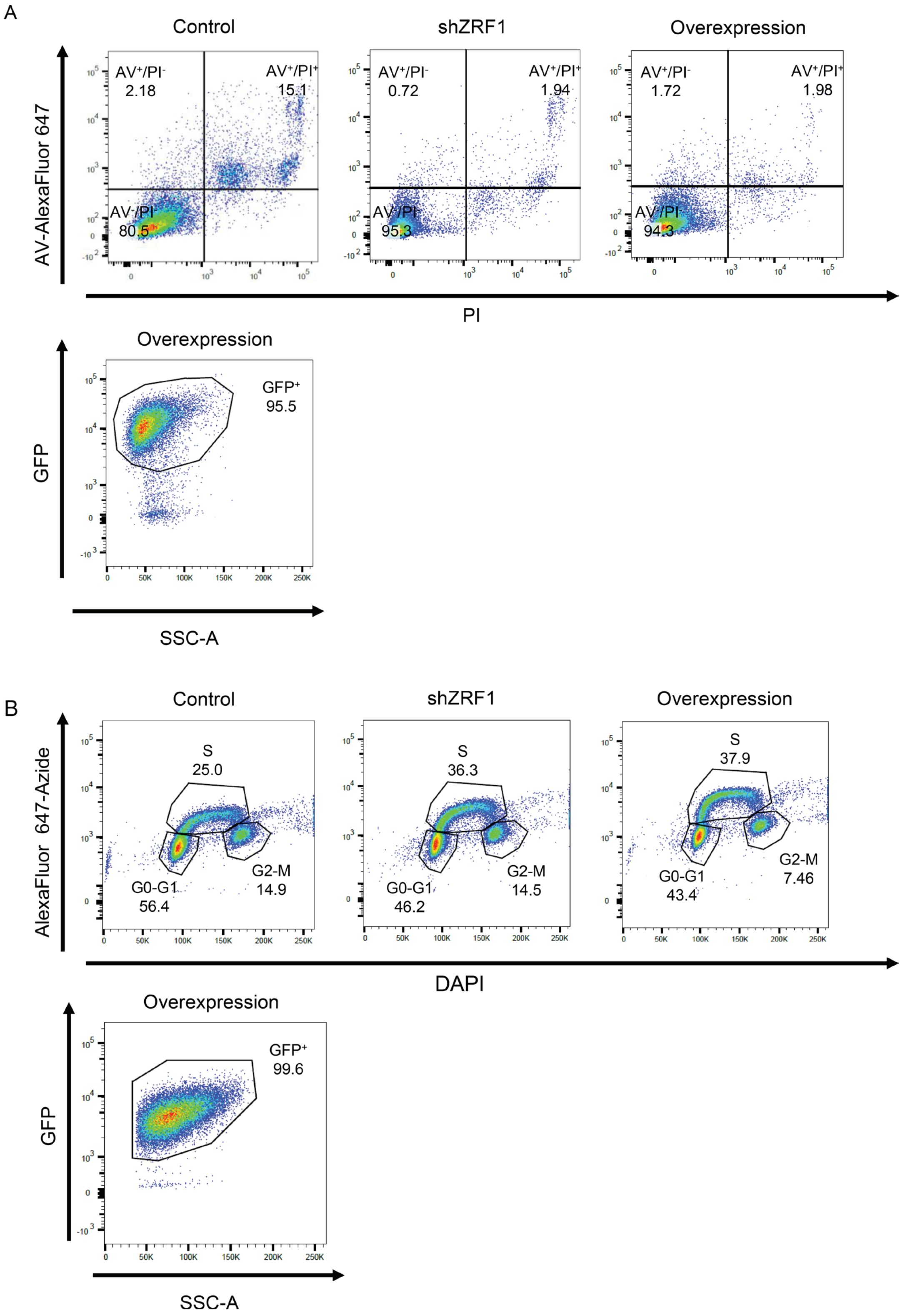

Supplementary Figure 10: Decreased cellular motility in ZRF1 overexpressing MDA-MB-231 cells is not related to decreased cellular proliferation. (**A**) Representative flow cytometry analysis of apoptotic cell distribution of control, shZRF1 and ZRF1 overexpression cells with AV-647 and PI double staining. AV^{+}/PI^{-}: Early apoptotic cells, AV^{+}/PI^{+} Late apoptotic cells, AV^{-}/PI^{-}: Viable cells. (**B**) Representative flow cytometry analysis of cell cycle distribution of control, shZRF1 and ZRF1 overexpressing cells after an iClick reaction together with DAPI staining.

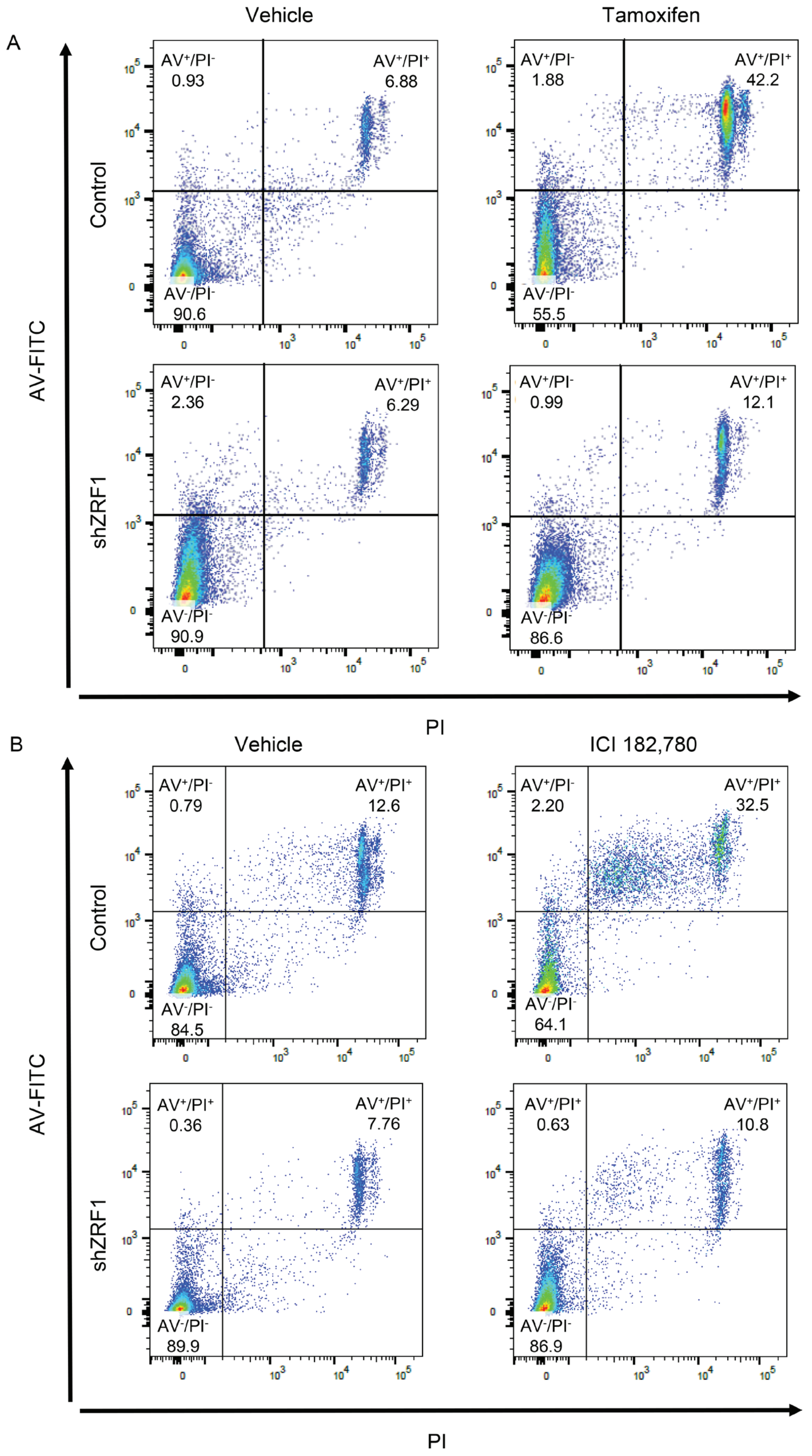

Supplementary Figure 11: ZRF1 depleted MCF7 cells are resistant to endocrine therapy. (**A**) Representative flow cytometry analysis of apoptotic cell distribution of control and shZRF1 cells after 48 hours of 10 μM Tamoxifen treatment with AV-FITC and PI double staining. AV$^+$/PI$^-$: Early apoptotic cells, AV$^+$/PI$^+$ Late apoptotic cells, AV$^-$/PI$^-$: Viable cells. (**B**) Representative flow cytometry analysis of apoptotic cell distribution of control and shZRF1 cells after 7 days of 100 nM ICI 182,780 treatment with AV-FITC and PI double staining. AV$^+$/PI$^-$: Early apoptotic cells, AV$^+$/PI$^+$: Late apoptotic cells, AV$^-$/PI$^-$: Viable cells.

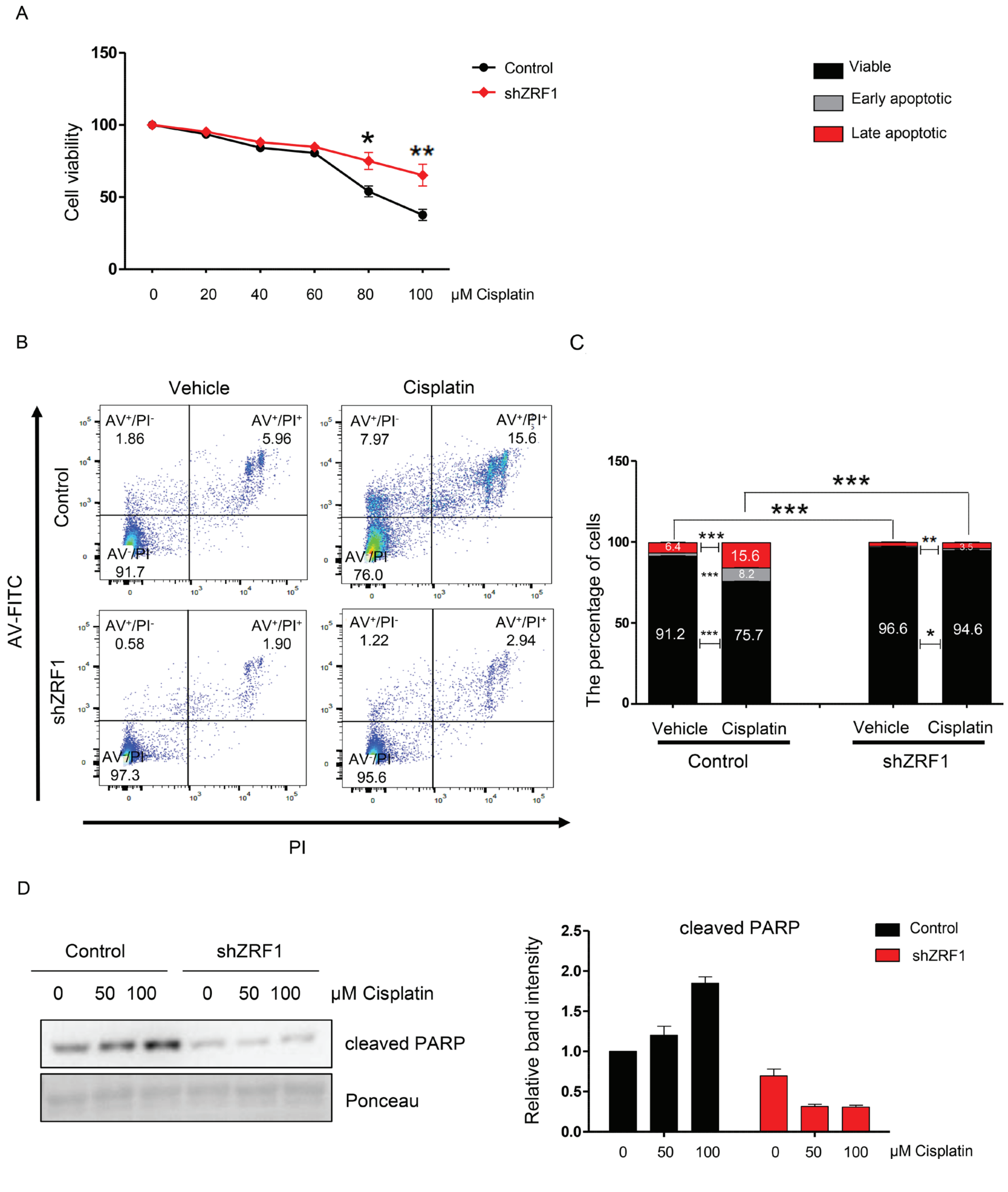

Supplementary Figure 12: ZRF1 depleted MDA-MB-231 cells are resistant to chemotherapy. (**A**) Cell viability measurements with control and ZRF1 depleted MDA-MB-231 cells after treatment with increasing doses of cisplatin employing MTT assays. Data represent the average of three experiments, ± S.E.M. $^{*}p < 0.5$, $^{**}p < 0.01$ as calculated by two-tailed unpaired *t* test between the samples indicated. (**B**) Representative flow cytometry analysis of apoptotic cell distribution of control and shZRF1 cells after 24 hours of Cisplatin treatment with AV-FITC and PI double staining. AV^{+}/PI^{-}: Early apoptotic cells, AV^{+}/PI^{+} Late apoptotic cells, AV^{-}/PI^{-}: Viable cells. (**C**) Flow cytometry analysis of apoptotic cell distribution of control and shZRF1 cells after Cisplatin treatment for 24 hours. Data represent the average of three experiments, ± S.E.M. $^{*}p < 0.5$, $^{**}p < 0.01$, $^{***}p < 0.001$ as calculated by two-tailed unpaired *t* test between the samples indicated. (**D**) Western blot of cleaved PARP protein in control and shZRF1 cells after administration of two different doses of Cisplatin for 24 hours. Ponceau was used as a loading control. Relative band intensity of each sample was calculated in relation to the control (0 hour band) intensity: (Relative Band Intensity of Cleaved PARP/ Relative Band Intensity of Ponceau).

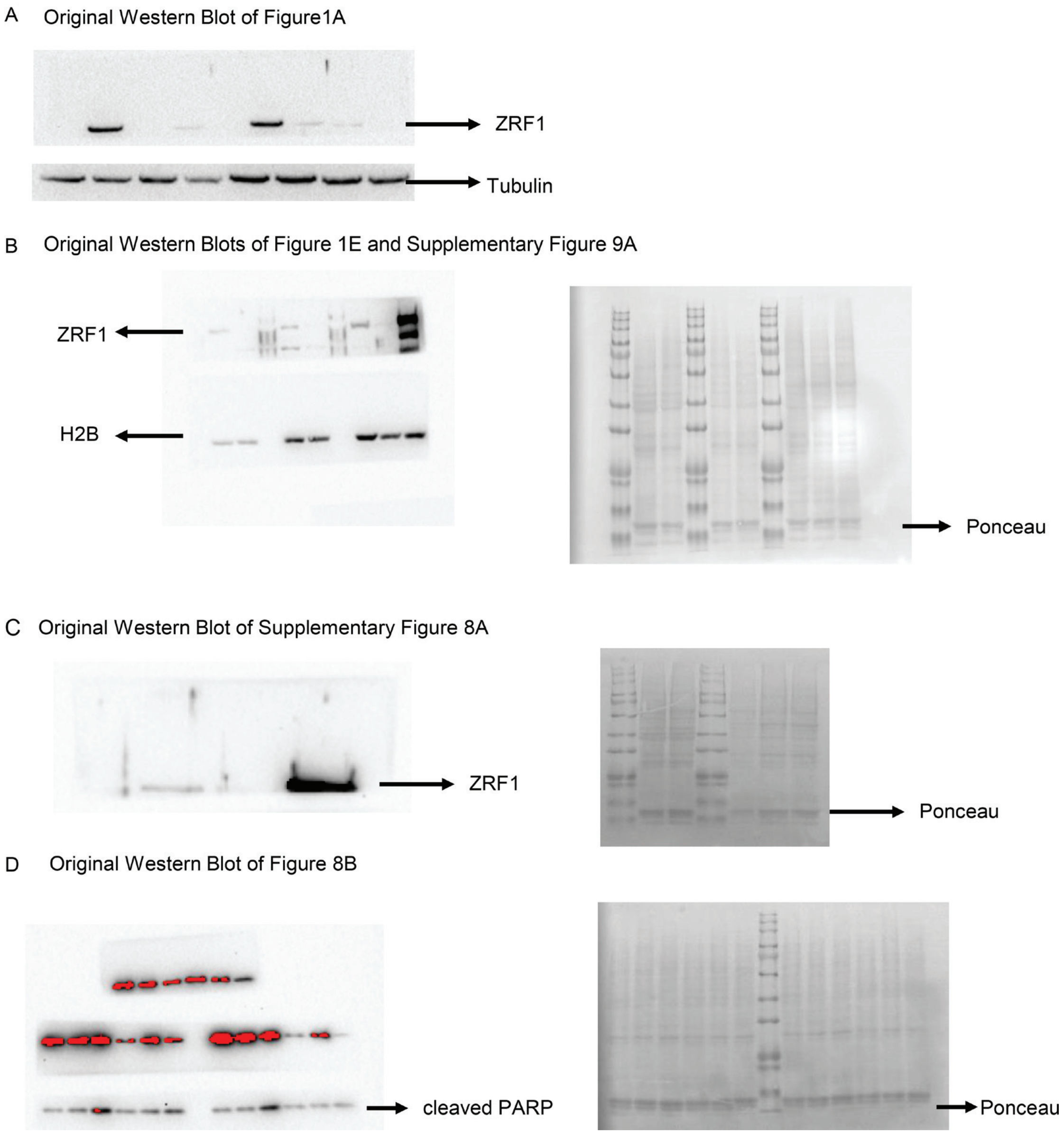

Supplementary Figure 13: Original Western Blot Figures. (**A**) Original western blot of Figure 1A. (**B**) Original western blots of Figure 1E and Supplementary Figure 9A. (**C**) Original western blot of Supplementary Figure 8A. (D) Original western blot of Figure 8B.

A Original Western Blot of Figure 8D

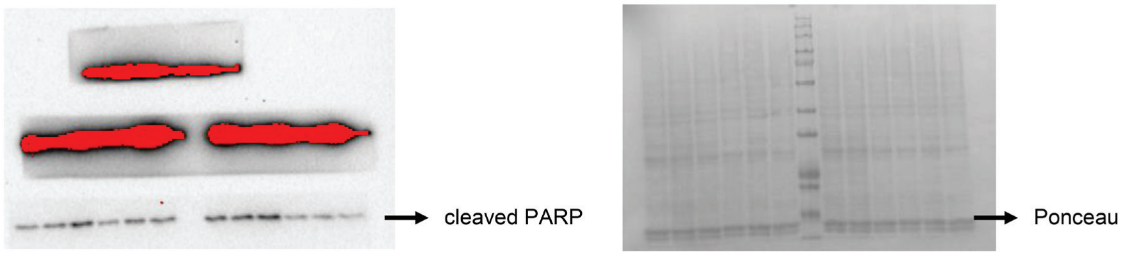

B Original Western Blot of Supplementary Figure 12D

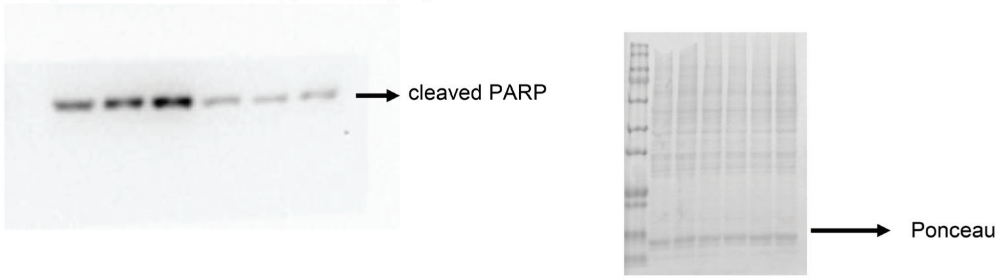

C Original Western Blot of Figure 9A

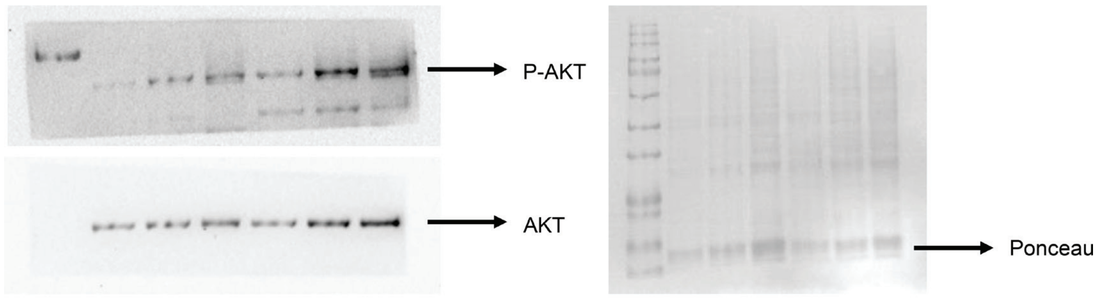

D Original Western Blot of Figure 9B

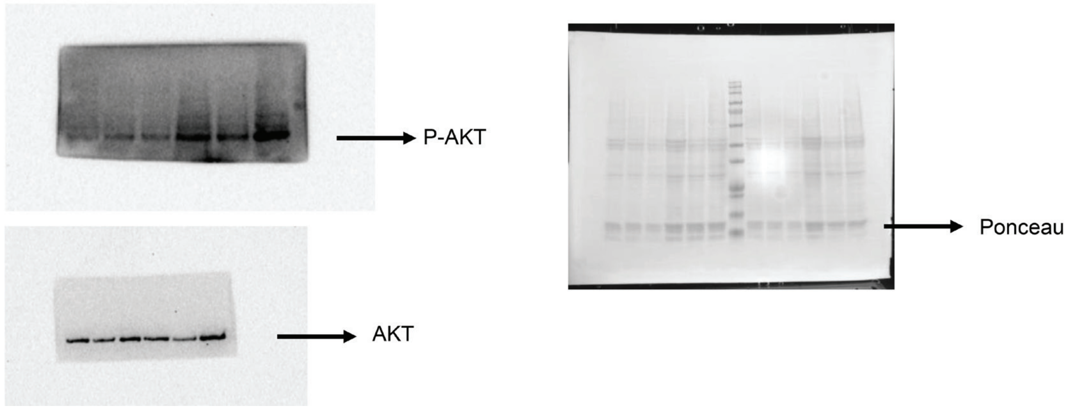

Supplementary Figure 14: Original Western Blot Figures-2. (**A**) Original western blot of Figure 8D. (**B**) Original western blot of Supplementary Figure 12D. (**C**) Original western blot of Figure 9A. (**D**) Original western blot of Figure 9B.

Supplementary Table 1: shRNA and mRNA primers. See Supplementary_Table_1

Supplementary Table 2: shZRF1_vs_shNMC (expression data from RNA seq). See Supplementary_Table_2

Supplementary Table 3: shZRF1_vs_shNMC_GO_KEGG_enrichment. See Supplementary_Table_3

Supplementary Table 4: FDR analysis. See Supplementary_Table_4
